# Glimmers of hope in large carnivore recoveries

**DOI:** 10.1038/s41598-022-13671-7

**Published:** 2022-07-21

**Authors:** Kurt E. Ingeman, Lily Z. Zhao, Christopher Wolf, David R. Williams, Amelia L. Ritger, William J. Ripple, Kai L. Kopecky, Erin M. Dillon, Bartholomew P. DiFiore, Joseph S. Curtis, Samantha R. Csik, An Bui, Adrian C. Stier

**Affiliations:** 1 Department of Ecology, Evolution, and Marine Biology, University of California, 2018 Noble Hall, Santa Barbara, CA 93106 USA; 2 David H. Smith Conservation Research Program, Society for Conservation Biology, Washington, DC USA; 3 Global Trophic Cascades Program, Forest Ecosystems and Society, Oregon State University, Corvallis, OR USA; 4 School of Earth and Environment, University of Leeds, Leeds, UK; 5 Bren School of Environmental Science and Management, University of California, Santa Barbara, CA USA

**Keywords:** Conservation biology, Ecology, Ecology, Environmental sciences

## Abstract

In the face of an accelerating extinction crisis, scientists must draw insights from successful conservation interventions to uncover promising strategies for reversing broader declines. Here, we synthesize cases of recovery from a list of 362 species of large carnivores, ecologically important species that function as terminal consumers in many ecological contexts. Large carnivores represent critical conservation targets that have experienced historical declines as a result of direct exploitation and habitat loss. We examine taxonomic and geographic variation in current extinction risk and recovery indices, identify conservation actions associated with positive outcomes, and reveal anthropogenic threats linked to ongoing declines. We find that fewer than 10% of global large carnivore populations are increasing, and only 12 species (3.3%) have experienced genuine improvement in extinction risk, mostly limited to recoveries among marine mammals. Recovery is associated with species legislation enacted at national and international levels, and with management of direct exploitation. Conversely, ongoing declines are robustly linked to threats that include habitat modification and human conflict. Applying lessons from cases of large carnivore recovery will be crucial for restoring intact ecosystems and maintaining the services they provide to humans.

## Introduction

Historically, the majority of conservation research has focused on critical cases where conservation objectives remain unmet^1^. An alternative approach seeks to borrow strategies from conservation successes^2,3^, using positive deviations, or ‘bright spots,’ to identify the factors influencing variation in recovery outcomes^4^. We apply this approach to large carnivores whose size and terminal position in food chains can lead to outsized ecological effects and conservation value^5^. Large carnivores, although taxonomically disparate, represent an ecologically coherent group^6^ as a result of the cascading effects they can have on ecosystems, including promoting biodiversity, altering nutrient cycling, and regulating disease^5,7^. Examining cases of large carnivore recovery and recolonization is critical for understanding the context-dependence of top-down effects^8^ and how ecosystem function compares among intact and defaunated ecosystems^9^. Although rebounding large carnivore populations can introduce new tradeoffs^10^, the socio-ecological benefits of their recovery often exceeds associated costs^11^. Yet the traits common among species at high trophic levels—large range requirements, low reproductive rates, and high potential for human-wildlife conflict—make large carnivore conservation particularly challenging and increase their vulnerability, especially as the proportion of landscapes and seascapes devoid of intense human impact continues to shrink^12^.

As a consequence of intense exploitation and widespread ecosystem transformation, the abundance of large carnivores globally has been dramatically reduced^13,14^. The exploitation of wildlife for human consumption represents a major source of food and income, particularly in rural regions^15^; accordingly, direct harvesting remains a primary threat to megafauna across ecosystems^16,17^. At present, 94% of freshwater megafauna are considered overexploited^18^ while oceanic shark populations have declined by more than two-thirds since 1970 due primarily to overfishing^17^. Beyond exploitation, agricultural expansion has led to steep declines in large carnivore populations, exemplified by the spread of oil palm plantations across Sumatran tiger habitat^19^. Furthermore, a growing number of large carnivore species are affected by emerging threats, including climate change and the bioaccumulation of organic pollutants^20^, which can act synergistically when climate-mediated loss of prey exacerbates contaminant exposure^21^.

Amid global large carnivore declines, however, recent work has documented a handful of recoveries that offer glimmers of hope. Among terrestrial predators, populations of wolves, bears, wolverines, and lynx across Europe have largely increased or remained stable in recent years despite high human population density in the region^22^. In the ocean, improved management and increasing protection of habitats have achieved notable predator recoveries^3^: the banning of gill nets and the protection of spawning aggregations have reversed declining population trends in Giant sea bass (*Stereolepis gigas*) in the North Pacific and Nassau grouper (*Epinephelus striatus*) in the Eastern Atlantic, respectively^23,24^. Examples such as these point to opportunities to scale-up and replicate successful strategies and offer a message of cautious optimism that recovery is possible—a message that is more likely to inspire action than the prevailing narrative of monolithic degradation^25^. Moreover, uncovering the conservation actions associated with these positive outliers is critical for moving toward evidence-based conservation^26,27^.

Here, we present a synthesis of large carnivore recoveries, examining the variation in global recovery outcomes and illustrating these results with concrete lessons from instructive cases. Using published databases, we assembled a list of large carnivore species, drawing from all major vertebrate groups and spanning ecosystems. We used criteria synthesized from the ecological literature pertaining to body size, diet, and ecology (see “Methods”) to assemble a list of large-bodied carnivores that function as a terminal consumer in a particular ecological context. We compiled data on population trends and extinction risk status from the International Union for the Conservation of Nature (IUCN) and examined how current extinction risk varies across the six major taxonomic groups represented: cartilaginous fishes, bony fishes, amphibians and reptiles, birds, terrestrial mammals, and marine mammals. We then assessed two temporal metrics indicative of recovery, (1) an increasing population trend, and (2) genuine improvements in IUCN extinction risk. We employed binomial logistic regression models to test associations between recoveries and conservation actions. Lastly, we examined the prevalence of anthropogenic threats affecting our species pool and tested their relationship with ongoing large carnivore declines.

### Patterns of extinction risk

We identified 362 vertebrate species distributed across all major ecosystem types (Fig. S1) that met our criteria and to which the IUCN has assigned a known extinction risk category. Overall, 38% [35%–43%] of large carnivore species are considered threatened (encompassing ‘Vulnerable’, ‘Endangered’, or ‘Critically Endangered’ species; lower and upper bounds reflect the uncertainty introduced by Data Deficient species). This represents a substantially higher proportion of threatened species compared to Red List averages of 27% for all species and 18% for all vertebrates. Among these species, extinction risk is unevenly distributed across geographic regions. Large carnivore species in the Nearctic and Australasia regions show a lower proportion of threatened species (13%, and 27% threatened, respectively); compared with the Afrotropic (39%) and Indo-Malay regions (41%). We note that developed regions with lower proportions of threatened species have previously lost sensitive megafauna to Pleistocene extinctions^28^, export a sizeable share of their environmental impacts^29^, and show lower rates of increase of human population compared with developing regions^30^.

**Figure 1:**
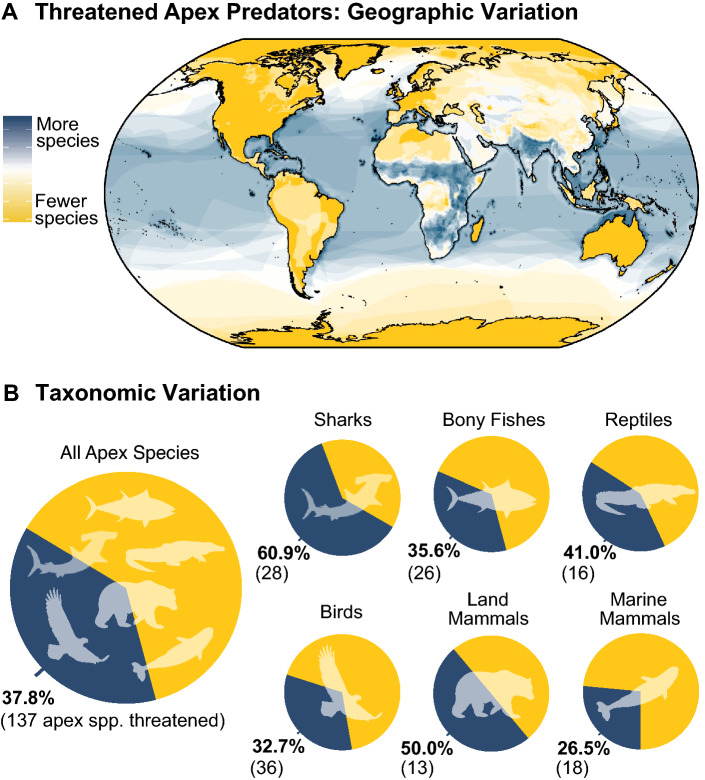
Variation in current large carnivore extinction risk (status). Large carnivores are divided taxonomically into sharks and rays, bony fishes, reptiles and amphibians, birds, terrestrial mammals, and marine mammals. Bold values are the percent threatened in each taxon; values in parentheses are the number of threatened species.

**Figure 2:**
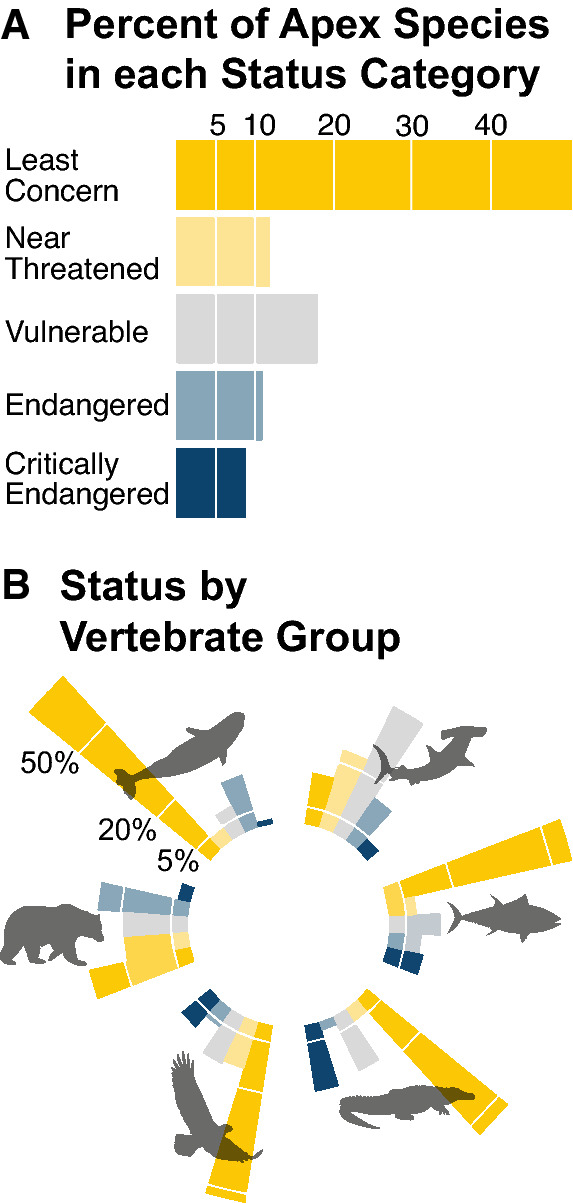
Current IUCN extinction risk. (**A**) Percent of large carnivore species that occupy each extinction risk category. Percentages calculated from species with known status only (i.e., ‘Data Deficient’ species omitted). Least Concern (LC) = 50.6%, Near Threatened (NT) = 11.6%, Vulnerable (VU) = 17.1%, Endangered (EN) = 11.0%, Critically Endangered (CR) = 9.7%. (**B**) Percent of species in each extinction risk category, split by major vertebrate group. Extinction risk categories colored as in A. Exact values for each taxon-status combination provided in Table S1.

Extinction risk also varied widely among vertebrate groups (Fig. 1). Marine mammals show the lowest proportion of threatened species (27% threatened [20%–44%]) offering a stark contrast with the high extinction risk among sharks and rays (61% threatened [55%–65%]). Indeed, while more than half of all large carnivore species are categorized Least Concern, only 17% of shark species occupy this lowest extinction risk category (Fig. 2; Table S1). The wide disparity in status for these two groups of marine megafauna corroborates evidence that while the cessation of industrial exploitation of marine mammals has initiated successful recoveries within this group^31^, many shark species remain intensely and unsustainably exploited^17,32^. As these groups of marine megafauna show little functional redundancy^33^, ongoing declines among sharks are likely to have deleterious ecological effects, including mesopredator release and altered carbon dynamics^34^. Among the remaining taxa, fully half of terrestrial mammals are listed as threatened, nearly double the rate of all IUCN mammal species. Further, roughly one-third of reptiles, birds, and bony fishes are threatened, compared with taxon-wide rates of 34%, 14%, 6% respectively.

### Large carnivore recoveries

**Figure 3:**
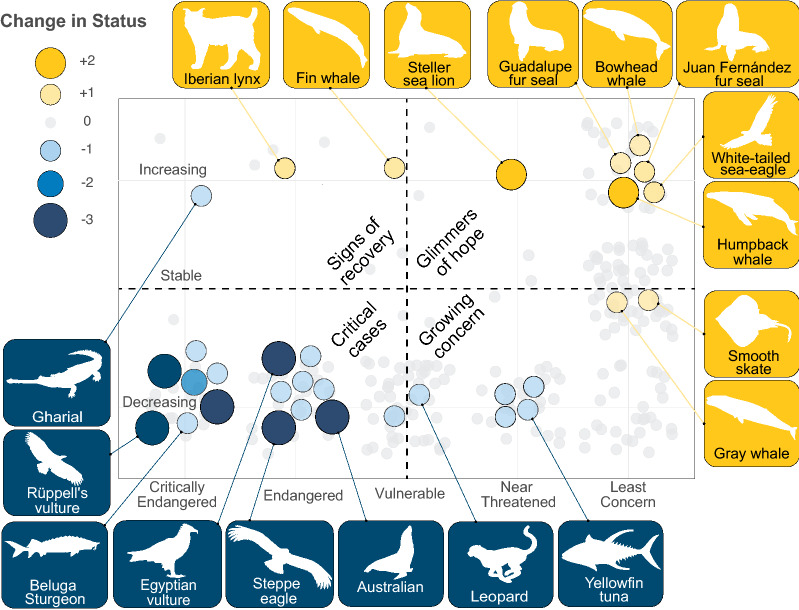
Glimmers of hope and critical cases. Distribution of large carnivore species across categories of current IUCN status (x-axis) and population trend (y-axis). Improvements in status are indicated by gold and declines by blue, with bubble size indicating the number of status category changes. The majority of species have not undergone any changes in status (shown in light gray). Note: No change in status may indicate lack of recent assessment, insufficient data, or, in the case of species designated Least Concern, effective conservation efforts.

On its own, current extinction risk provides only a snapshot of conservation status. We therefore assessed two temporal metrics indicative of recovery: (1) an IUCN-listed ‘increasing’ population trend and (2) a cumulative improvement in status since the species’ first comprehensive assessment under the modern IUCN system (ranging from 1996 to 2001, depending on taxon). Importantly, we considered only genuine improvements that came as a result of conservation efforts, excluding changes that resulted from improved assessment data or from IUCN criteria changes^35^. Approximately 10% of large carnivores (39 species) displayed one or both indicators of recovery, and recoveries were highly concentrated within particular vertebrate groups, including birds and marine mammals. Marine mammals showed higher than expected number of species with both increasing trend (p > 0.001; Bonferroni-corrected, post-hoc binomial test; Table S2) and genuine status improvement (p > 0.001), highlighted by dramatic improvements in status of humpback whales (*Megaptera novaeangliae*) and Steller sea lions (*Eumetopias jubatus*) (Fig. 3). Few genuine status improvements fell outside of the marine mammals and no other vertebrate group showed higher than expected number of species with either recovery metric (Table S2). Baleen whales alone represented nearly 18% of all recoveries and, omitting marine mammals, the proportion of recoveries among remaining taxa dropped to 7.3% (21 of 294 species). While birds showed a number of species with increasing trend (n = 14; Table S4), many species in this group remain in peril. We identified only a single terrestrial mammal, the Iberian lynx (*Lynx pardinus*), that met either recovery criterion.

Recovery was similarly uneven with respect to geography (Table S3; Figs. S2–S3). Compared to expected proportions based on the global prevalence of large carnivore predator recovery, the Nearctic realm had a significantly greater proportion of species with an increasing population trend (47% increasing, p < 0.001, Bonferroni-corrected, post-hoc binomial test) and with improved IUCN status (7% improved, p < 0.001). In contrast, the Afrotropic and Indo-Malay realms showed significantly higher than expected proportions of species with a decreasing trend (62%, p = 0.008 and 72%, p < 0.001, respectively) and status declines (18% and 7%, p < 0.001 for both). Among marine realms, several temperate and polar regions showed greater than expected proportions of species with improvements in status (Northern Temperate and Arctic Oceans, 7%; Northwest Pacific, 5%; Southeast Pacific 7%, and Southern Ocean, 10%; p < 0.001 for each), driven largely by marine mammal recoveries.

### Conservation strategies associated with recovery

**Figure 4:**
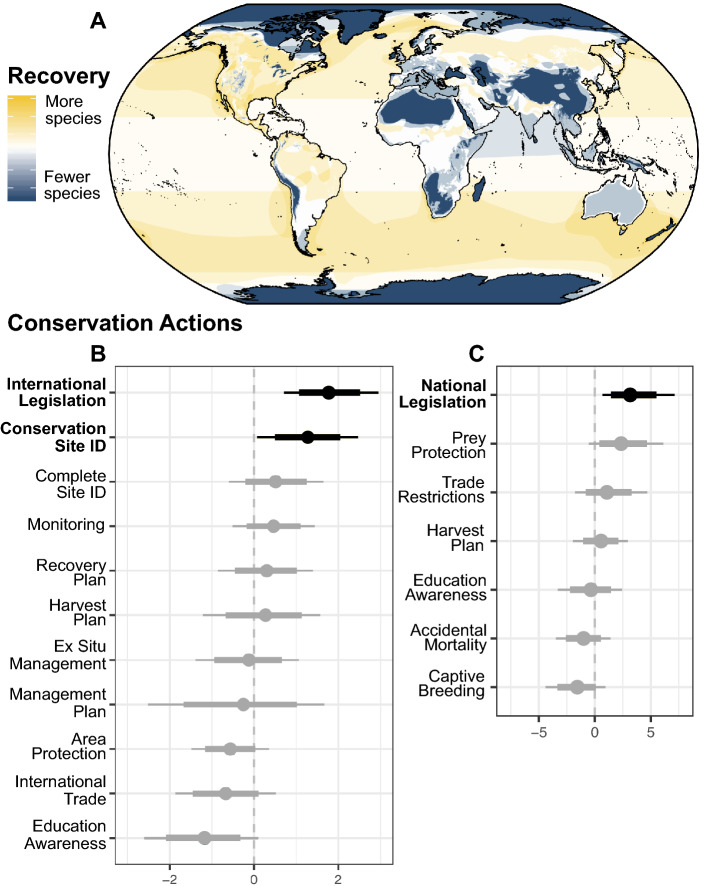
Large carnivore recoveries and associations with conservation actions. (**A**) Geographic variation in the number of large carnivore recoveries, defined as an increasing population trend or improvement in status since initial IUCN assessment (cumulatively, *recovery metrics*). IUCN range maps of species meeting either criterion were overlaid, and color indicates the absolute number of recovering species in each 5 km cell. (**B**) For IUCN-defined ‘Conservation actions in-place,’ the change in the log-odds of showing an increasing trend for species subject to each conservation action (see Table S5 for definitions). Points to the right of the dashed vertical line (black) indicate a significant positive association, Gray points indicate that confidence intervals overlap zero. Parameter estimates are coefficients of a binomial logistic regression model (with all other conservation actions included), 95% confidence intervals (thin lines), and 90% confidence intervals (thick lines). (**C**) For author-defined conservation actions (see “Methods”, Table S5), the change in the log-odds of a positive outcome of either recovery metrics. Not shown: IUCN-defined actions and improved status (significant positive associations: International Legislation and Harvest Plan).

This variation in recovery outcomes allowed us to explore the categories of conservation interventions (Table S5) positively associated with large carnivore recovery using binomial logistic regression. Recoveries were associated with species legislation enacted at national and international levels, and with harvest plans that reduce uncontrolled exploitation (Fig. 4). Specifically, the odds of an improvement in extinction risk increased 6.8-fold [CI 1.9–122.9] for species subject to international legislation and threefold [CI 1.8–45.6] for species with a harvest management plan in place (using IUCN-defined categories; Table S5). Similarly, the odds of an increasing population trend more than doubled for species subject to international legislation and those with conservation sites identified (Fig. 4b). Under IUCN guidance, conservation sites identified may or may be receiving protection (see Table S5 for details) and this action should not be confused with area protection, which was not associated with either indicator of recovery. We note two important caveats. First, among IUCN Red List assessments, ‘Conservation Actions In-place’ fields are inconsistently completed (IUCN Red List, pers comms.). Second, several species with near total harvest bans (e.g., marine mammals) are listed as having harvest management plan in-place, likely driving the association with recovery. Indeed, with marine mammals omitted from the analysis, harvest management plans did not significantly increase the odds of a status improvement (CI − 2.1–+ 3.9). In response to these limitations of IUCN-defined categories, the authors compiled a separate list of conservation interventions applied to large carnivores (see “Methods” and Table S5) and further tested associations with recovery metrics. From models using author-defined conservation categories, species with national legislation were 13 times more likely to have a positive than a negative recovery outcome [CI 1.9–122.9] (Fig. 4c).

Our analysis underlines evidence that legal protections at the national level can be instrumental in facilitating wildlife recoveries. Notably, recoveries were disproportionately found in developed regions where the application of strong legislation and management of exploitation is made possible by effective governance and enforcement. For example, the eastern population of Steller sea lions (*Eumetopias jubatus*) has increased 2% per year between 2000 and 2015 as a result of strong national legislation, which has provided the leverage to reduce direct mortality and to identify and protect critical rookery habitat^36^. This example corroborates broader evidence that strongly enforced limits on direct mortality coupled with the identification of critical habitat enhances recovery^37^ and offers a timely reminder of the value of national legislation as the United States emerges from a period of pronounced deregulation^38^.

Legal protections have also been crucial for the recovery of high-trophic level birds, from the regulation of the insecticide *DDT* (banned in 1972 in the United States and Canada; most western European countries in the 1980s) to more recent agreements^39,40^. Yet despite regulations, large avian carnivores remain vulnerable, as demonstrated by the catastrophic declines of South Asian vultures, driven largely by the use of the non-steroidal anti-inflammatory drug (NSAID) *diclofenac* to treat livestock in this region^41^. Moreover, the recent licensing of *diclofenac* for veterinary use in several southern European countries could lead to thousands of vulture deaths a year^42^, potentially undermining a regional bright spot in our analysis—the Iberian Peninsula. More encouragingly, conservation research on South Asian vultures has progressed from identifying primary threats to designing, implementing, and testing effective interventions (including safe alternatives to *diclofenac*); ongoing monitoring suggests shallower declines and even the incipient recovery of some vulture populations^43^. These contrasting case studies demonstrate the importance of enforceable regulation at the national level, and of providing alternatives to prohibited activities.

In contrast, we found no support for a positive effect of area protection (i.e., parks and refuges), a result that should be interpreted cautiously and with context. Considerable evidence suggests that protected areas can be an effective tool for conserving megafauna on land^44^ and in the ocean^45^. Yet, many parks are poorly enforced (‘paper parks’) with exhibit low levels of compliance with park restrictions^46,47^, reducing their effectiveness in achieving conservation aims^48^. The lack of detectable effects of area protection on recoveries may also stem from inadequate size and lack of connectivity with habitats outside of parks^49^ as even large protected areas offer only partial refuge for highly mobile predators^50^.Thus, our results reinforce the message that area protection should be accompanied by addressing conditions that enable their success—within and beyond park boundaries^51^.

### Reversing ongoing declines

**Figure 5:**
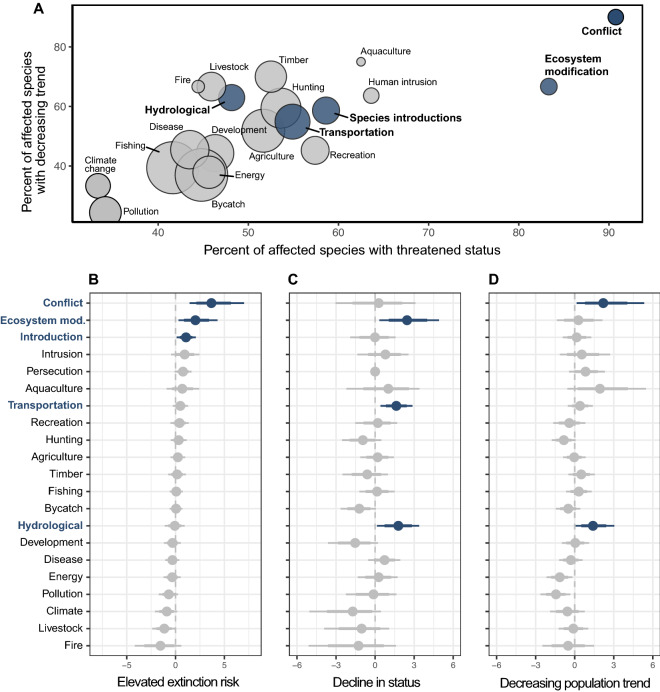
Ongoing declines and associations with anthropogenic sources of threat. (**A**) 23 anthropogenic threats (see descriptions in Table S6) plotted according to the percent of species affected by the threat with threatened status (x-axis) and with decreasing population trend (y-axis). The size of each point indicates the absolute number of species affected, ranging from fishing (n = 128) and bycatch (n = 125) to fire and aquaculture (n = 9 and 8, respectively). Points in blue are associated with at least one metric of decline. (**B**) The change in the log-odds of showing elevated extinction risk for species subject to each anthropogenic threat. Points to the right of the dashed vertical line (blue) indicate a significant positive association. Gray points indicate that confidence intervals overlap zero. Parameter estimates are coefficients of a binomial logistic regression model (with all other threats included), 95% confidence intervals (thin lines), and 90% confidence intervals (thick lines). (**C**) Associations between threats and decline in status (increasing extinction risk through time). (**D**) Associations between threats and a decreasing population trend.

Complementing our analysis of conservation actions associated with recovery, the negative outcomes in the data also enabled us to identify anthropogenic threats associated with ongoing declines. We tested whether 23 categories of threat (Tables S6 and S7) were associated with any of the three negative outcomes: high extinction risk, declines in status through time, and decreasing population trend. Among these, five threats were significantly linked with negative outcomes (Fig. 5) and three of these (conflict, ecosystem modification, and hydrological modification) were associated with multiple metrics of decline. Specifically, species that whose range included conflict zones (“conflict” here denotes war or instability, rather than human-wildlife conflict) had a 37-fold increase in the odds of elevated extinction risk [CI 4.6–890] and a 7.4-fold increase in the odds of a decreasing population trend [CI 1.3–142]. We note that the effects of conflict may disproportionately affect terrestrial megafauna; consequences for the conservation of large marine predators remains relatively understudied and merits future research. Recent work has documented the corrosive effect of armed conflict on the stable governance and civil institutions critical for conservation, and our findings indicate that the links between warfare and wildlife declines observed in African parks^52^ may hold for large carnivores globally. Such instability may emerge as an even greater threat to large carnivore recovery in the near future, given growing human populations, the global rise in income inequality, and the potential for climate change to exacerbate resource conflicts. Therefore, scalable and rapid-response interventions that simultaneously alleviate the effects of conflict on humans and wildlife will become increasingly critical. Encouragingly, there is some evidence that recovery remains possible even in regions of frequent conflict given timely, post-conflict interventions—particularly those that link wildlife rehabilitation to poverty alleviation^53^.

More broadly, effective predator conservation in the developing world may require an alternative model to that of developed nations: given the competing pressures of poverty and other social concerns, collaborative approaches emphasizing local engagement, psychological ownership, and capacity building may be more effective than top-down, legislative approaches^54^. Incentive-based strategies have significantly reduced retaliatory killings of African lions (*Panthera leo*) in Maasailand in Southern Kenya by compensating pastoralists for depredated livestock, and by employing respected community members to reinforce predator-tolerance behaviors and encourage a sense of community ownership for predators^55^. While education and awareness approaches were not associated with recoveries in our global analysis, examples such as this highlight the efficacy of well-designed social interventions in rural and developing regions.

In addition, ecosystem modification was linked with elevated extinction risk [8.9-fold increase; CI 1.7–84] and status decline [5.6-fold increase; CI 1.9–29] and hydrological changes (e.g., dam construction) were associated with declining status [4.0-fold increase; CI 1.1–14] and with decreasing trend [4.5-fold increase; CI 1.1–14]. These threats are particularly acute for fishes in Asia and Africa where construction of dams^56^ and conversion of mangrove habitat^57^ is accelerating. Accordingly, bony fishes in the combined Indo-Malay and Afrotropic regions showed the highest proportion of threatened species among all taxon/region combinations in our analysis (44%). This result highlights the urgency of conservation of freshwater and estuarine habitats being squeezed by the synergistic effects of exploitation, development, and changing climate patterns^58^.

Among other taxon/region combinations, the Indo-Malay and Afrotropic birds were disproportionately represented among species experiencing ongoing declines, in particular African eagles and vultures (Fig. 3, Table S4). Declines among these scavengers has been attributed to inadvertent poisoning (when vultures consume poisoned baits targeting predators), trade in body parts for medicinal use, and direct persecution by poachers (due to their role in signaling authorities of illicit activity)^59,60^. Globally, dietary toxins represents the most prevalent threat facing vultures^61^, but it is notable that scavengers—uniquely among the species in our analysis—rely on provisioning of carrion by predators and may therefore be subject to the compounding effect of declines in other large carnivores from our study (“chain of extinction”). While more difficult to quantify, the impact of wildlife declines across Africa has likely affected vulture populations, in turn^60^, highlighting the critical importance of large carnivore recovery. Indeed, with scavenging species omitted, the association between ecosystem modification and negative outcomes was no longer significant (Table S8), perhaps reflecting indirect effects on vultures mediated through habitat- and prey-loss for the predators on which they rely. Even more directly, the association between status declines and transportation disappeared with vultures omitted, underling the vulnerability of scavenging species to road-strikes.

## Discussion

Large carnivore recovery represents a unique opportunity for reversing broader degradation of ecosystems. As some of nature’s most charismatic species, many serve as national symbols and flagship species whose conservation can indirectly benefit other species and increase support for about conservation initiatives^62^. Our global synthesis documents rare cases of recovery among large carnivore species and underlines the importance of species legislation and restricting or eliminating exploitation for recovery success. While the utility of these conservation actions has been previously demonstrated in finer-scale analyses, our work emphasizes the generality of these findings for large carnivores globally and provides an important cross-taxon comparison of their implementation. We further uncovered robust links between ongoing declines and specific anthropogenic threats, including human conflict, habitat modification, and hydrological changes. A core message that emerges is that well-enforced legislation and management of exploitation can underpin large carnivore recovery by addressing uncontrolled mortality and habitat degradation that can occur in the absence of effective governance. Our findings illuminate large-scale patterns that can serve as hypotheses for exploring the variation in recovery within species and among populations and are best viewed as a complement to important and ongoing species-specific and regional analyses^63^. A detailed policy review of specific legal provisions, the extent of enforcement, and the resulting magnitude of recovery is beyond the scope of our analysis but represents an important future direction. Nevertheless, it is instructive that these results have emerged from a global, cross-taxon analysis despite the inherent variability in such an effort.

Examining rare glimmers of hope among large carnivore recoveries can highlight strategies for reversing population declines that remain more common within this group. Recoveries that buck taxonomic trends are instructive as exceptions that illuminate the rule: although recovery for sharks as a whole was not encouraging, successful rebuilding of elasmobranch populations has occurred in the small number of cases where exploitation limits are grounded in science and strictly enforced^17,64^. Indeed, one of the bright spots from our analysis, the smooth skate (*Dipturus innominatus*) owes its recovery to inclusion in New Zealand’s quota management system, which has resulted in newly sustainable harvest levels^65^. We caution against inferring from this example that sustainable exploitation is achievable for all large carnivore species, however. Factors including K-selected life-history traits and social group dynamics can mean that even modest levels of exploitation can result in large carnivore population declines^66^ and exploitation policies must align with ecological realities.

Recoveries in human-occupied landscapes are particularly encouraging and instructive. For example, the Spanish imperial eagle (*Aquila adalberti*) declined to near extinction on the intensively altered Iberian Peninsula as a result of ecosystem modification and resultant loss of prey, as well as inadvertent poisoning and electrocution^67^. Strong species recovery since the 2000s, largely outside of protected areas, has been attributed to the replacement of electrical infrastructure and to programs supporting eagle-friendly land management and prey protection on private lands^68^. This example demonstrates that large carnivore declines can be reversed even in regions with limited public lands by addressing critical threats in partnership with private landowners.

Many large carnivore species have vast ranges encompassing several countries, complicating both the implementation of conservation interventions and the assessment of their effectiveness. For example, globally, tigers (*Panthera tigris*) have been extirpated from 94% of their historic range^69^ and remain Endangered with a decreasing population trend. Yet regional, national, and landscape-scale interventions are showing promise for tiger recovery: an ambitious program involving all 13 tiger-occupied countries are underway with the goal of doubling the global population through preserving habitat, eradicating poaching and illegal trading, and engaging with local communities^70^. Indeed, Nepal has nearly doubled its Bengal tiger population since 2009 through locally tailored efforts aimed at reducing human-wildlife conflict and supporting the well-being of communities that share landscapes with tigers^71^.

For the many species whose home range spans multiple jurisdictions, regulating exploitation and protecting sensitive habitats requires consistency and cooperation across state/territorial and international borders^39,40^. Accordingly, international protections also showed a positive association with successful large carnivore recovery, exemplified by the moratorium on commercial whaling^31^. The future success of international agreements aimed at large carnivore recovery will rely on responsive trans-jurisdictional governance, especially as species ranges’ shift with climate change^72^. At present, negotiations are underway for a UN treaty on conservation of the ‘high-seas’ (marine regions that fall outside any national jurisdictions), an agreement that is particularly critical for reversing ongoing declines in pelagic sharks and migratory tunas (Fig. 2).

Our analysis also revealed important data gaps. Despite the ecological and symbolic importance of large carnivore, only 65% of assessed species were assigned to definitive status and trend categories: 33 species were considered Data Deficient, including iconic predators such as the Killer whale (*Orcinus orca*) and an additional 103 assessed species had an unknown population trend. Moreover, many assessments are quite dated, limiting their utility for assessing real-time extinction risk and instantaneous population trends (while remaining useful for an analysis of historical recoveries and declines, as presented here). At the time of writing, 25% of assessments had not been updated for over a decade, with reptile assessments being particularly outdated (median age 9.6 years; Fig. S4). These gaps highlight the ongoing shortfall in investment in research and monitoring of wildlife populations—even among charismatic species that are widely perceived by the public to be well-studied and adequately protected^73^.

### A sustainable future: large carnivore recoveries in human-nature systems

Looking ahead, Anthropocene recovery strategies for large carnivores must be effective not only in largely unaltered landscapes, but also where ecosystems simultaneously sustain human livelihoods. It is in these shared landscapes and seascapes that the potential downsides of large carnivore recovery for humans are most acute^74^. Positive steps forward include maximizing social tolerance for and coexistence with predators^75,76^, as well as developing low-cost methods of reducing human risk^77^. Conversely, segregating human and carnivore activities^78^ and erecting physical barriers^79^ represent promising alternatives to culling, particularly when both human and predator safety is integrated into the design^80^.

Direct harvesting for human consumption or medicinal uses has historically been the greatest individual threat to megafauna across major classes of large terrestrial, freshwater, and marine vertebrates, and this overexploitation remains a major impediment to megafauna recoveries^16^. Moreover, new challenges are also emerging (e.g., climate change, resource conflicts) that may undermine previously successful efforts to address historical threats^81^. To adapt to this rapidly changing landscape for recovery, conservation strategies must merge forward-looking legislation to protect species with responsive resource management^82,83^. There is no silver bullet to reverse species declines, but successful efforts to recover populations must look beyond narrowly addressing conservation threats toward enabling the conditions for recovery in the context of sustainable human-nature systems.

## Methods

### Large carnivore species list

To build a comprehensive species list, we combined species trait databases developed for amniotes (Myrvold et al. 2015; n = 21,323) and vertebrates (Ripple et al. 2017; n = 27,647) and refined the list based on taxon- and ecosystem-specific criteria to retain a list of large carnivores. Specifically, we pooled candidate species from all vertebrate classes, pruning the list by (1) eliminating exclusively herbivorous families, (2) employing taxon-specific body mass thresholds drawn from previous literature, and (3) retaining only species whose primary dietary mode is carnivory. We restricted our list to species above the following taxon-specific body mass thresholds, following or approximating previously published large-carnivore size cutoffs where possible: Sharks and rays, marine teleost, marine mammal: 50 kg (Barnosky 2008); Freshwater teleosts, 20 kg (He et al. 2019); Reptiles and Amphibians: 10 kg (McClenachan et al. 2016); Birds: 1 kg; Terrestrial mammals: 12 kg (Ripple et al. 2016). To narrow the resulting large vertebrate list further, we queried FishBase using the package rFishBase (v17.07) in the R statistical environment (version 1.2.1268) and restricted sharks, rays, and bony fishes to those with a mean trophic position (‘DietTroph’) of 4.0 or greater. For species not available in rFishBase (n = 155) we compiled an equivalent metric of trophic position from a database published by Sánchez‐Hernández and Amundsen^84^. We acknowledge the limitations inherent in estimates of trophic position, including uneven sampling among species, seasonal variability, and ontogenetic diet shifts^84^ and recognize that improved diet data could refine future species lists. To remove mammalian herbivores and omnivores with plant-centric diets, we retained only species identified by^85^ as carnivores and, using the Phylacine 1.2 database^86^, we removed species with less than 50% carnivory listed.

We queried the IUCN Red List database (v2019.3) and compiled current information on species-level extinction risk (*status*: Not Evaluated, Data Deficient, Least Concern, Near Threatened, Vulnerable, Endangered, Critically Endangered, Extinct in the Wild, Extinct), population trend (*trend*: increasing, decreasing, stable), ecosystem type, and geographic range. Except where noted, we analyzed only species with known status (i.e., not Data Deficient). We calculated the average age of most recent assessment for our species list and compared age of assessment metrics across vertebrate groups with a Kruskal–Wallis one-way analysis of variance, using the function kruskal.test() from the R package *stats*. We found a significant difference in age-of-assessment across groups (chi-squared = 159.4, df = 5, p < 0.001). We then used the function dunnTest from the R package *FSA* to perform post-hoc tests while accounting for multiple comparisons and found significant differences among all groups, with the exceptions of comparisons between reptiles/amphibians and bony fish, as well as between marine and terrestrial mammals (Fig. S3).

### Species richness and range maps

We estimated large carnivore species richness using IUCN Red List and BirdLife species range maps (BirdLife International and Handbook of the Birds of the World 2016; IUCN 2018). For each species’ range map, we removed all polygons except those with “Origin” coded as either Native or Reintroduced and “Presence” coded as either Extant or Probably Extant. We then rasterized each species range at 5 km resolution. For species with altitude limits published in the IUCN Red List, we masked out areas outside these limits using the U.S. Geological Survey’s Global 30 Arc-Second Elevation map (re-projected to 5 km). We summed up the individual species’ raster range maps to form total large carnivore species richness. For mapping, we used separate scales to distinguish among terrestrial marine species richness, but combined freshwater and terrestrial species for visualization purposes. Because species may either lack spatial data or be classified as, for example, Possibly Extinct throughout their ranges, some of the species were not included in the richness map. In total, 9.4% (34/362) of the large carnivore species were excluded (Fig. S1).

We overlaid species range maps onto maps of marine biogeographic realms^87^ and terrestrial realms (for both terrestrial and freshwater species)^88^. For each species, we calculated the area of its range within each realm. We also calculated the proportion of its range within each region realm within Marine and Terrestrial realm types. We masked out marine realms in the small coastal areas where the terrestrial and marine realm maps overlapped. We reported marine realms for species that are at least partly marine (according to IUCN Red List fact sheet pages) and terrestrial realms for species that are at least partly terrestrial or freshwater. Note: given known limitations of marine species range identification, terrestrial range maps likely offer greater accuracy with respect to small-scale species richness patterns.

### Taxonomic and geographic patterns of extinction risk and recovery

We tested whether each taxon (Table S2) or geographic region (Table S3) showed a disproportionate prevalence of recoveries (or ongoing declines) using chi-square goodness-of-fit tests. We tested whether the proportions of each population trend category/level (*increasing*, *decreasing*, *stable*) for each (1) taxon and (2) region/realm differed from expected as well as whether the proportions of each change in status category/level (*improved*, *declined*, *unchanged*) for each (3) taxon and (4) region/realm differed from expected.

We informed the null expected distribution of outcomes using IUCN Red List data from 2007 to 2018. To inform the expected distributions of population trends across taxa (analysis 1), we calculated the proportion of each population trend category (*increasing*, *decreasing*, *stable*) across all assessed species belonging to each taxon. To inform the expected distributions of change-in-status across taxa (analysis 3), we compiled all genuine changes (see above) and calculated the proportion of each change-in-status level (*improved*, *declined*, *unchanged*) across all assessed species belonging to each taxon.

Finally, the proportions of population trend and change-in-status levels for all assessed species were tallied across all realms and used to inform the null expected distribution for the regional analyses (analyses 2 and 4 above, respectively). Most of these distributions were leptokurtic, with many species remaining unchanged. When the expected values were < 5, we obtained simulated p-values using Monte Carlo simulation. Significant chi-square goodness-of-fit tests were followed by post-hoc binomial tests with sequential Bonferroni corrections to obtain p-values for each specific outcome (e.g., Does the proportion of birds with decreasing population trend differ from expected?). We also conducted chi-square tests of independence for comparisons 1–4 above (e.g., Are taxon and population trend independent?). When expected values were < 5, p-values were obtained using Fisher’s Exact Test.

### Associations with recovery and ongoing declines

To examine recovery outcomes and their association with threats and conservation actions, we used multiple recovery metrics including population trend from the most recent assessment and change in status over time. For the latter, we obtained a complete history of IUCN Red List assessments and calculated the magnitude and direction of most recent change status (e.g., Vulnerable to Least Concern: + 2) and the cumulative change in status. Following^89^ we limited changes in status to those that have occurred since each taxonomic group was comprehensively assessed (Mammals, 1996; Birds, 1988; Reptiles, 1980) or 1996 for all other taxa. To evaluate only genuine changes in extinction risk as a result of improvement or declines in conservation outlook (as opposed to status changes that resulted in updated population information) we referenced Table S6 of genuine changes in^35^ (for changes prior to 2007) and annual lists of genuine and non-genuine changes provided by IUCN in Summary Statistics, Table 7 (2007–2019). Where species underwent multiple status changes, we calculated status change metrics using only genuine changes, making our results qualitatively similar to calculation of the Red List Index (RLI) for taxa that have undergone comprehensive assessment. We note that these recovery metrics are conservative indicators; change in extinction risk status, in particular, represents a high bar for recovery as ‘no change’ may indicate an absence of data or outdated assessment.

To evaluate drivers of recovery, we compiled information on conservation actions from the most recent IUCN assessments and compared patterns of actions employed for species with positive versus negative recovery outcomes. To do so, we compiled data for each species using the 11 IUCN-defined categories under ‘Conservation Actions In-Place’ (e.g., International Legislation) (Table S5). We then tested whether conservation actions were significantly associated with positive recovery outcomes using binomial logistic regression, where responses were positive only when status had ‘improved’ (merging ‘unchanged’ with ‘declined’ for this analysis) or when trend was ‘increasing’ (merging ‘stable’ with ‘decreasing’). We employed backwards stepwise selection to identify the best performing binomial logistic regression model with respect to AIC and used the coefficients of the resulting model to infer the change in log-odds of recovery associated with each action. Because recoveries were dominated by marine mammals, we repeated the analysis with and without this taxonomic group in order to identify associations between recoveries and conservation actions that were attributable to this group alone.

To complement IUCN-defined conservation actions, we created a list of 15 common conservation actions (nested in 5 broader categories; see Table S5) based on the text of the IUCN assessment “Conservation Actions In Detail.” We then tested whether conservation actions were significantly associated with positive recovery outcomes using binomial logistic regression, with statistical procedures identical to those described above for IUCN-defined categories.

We tested associations between IUCN listed threats and ongoing declines using binomial logistic regression. The Red List has well-known issues with its categorization of ‘threats’ and ‘stresses’ which conflate the sources of and responses to species declines, and which result in non-intuitive groupings (e.g., timber and hunting receiving the same ‘threat’ coding). We therefore re-categorized IUCN information into 23 author-generated categories of anthropogenic threat representing the relevant sector or human activity threatening the focal species (Tables S5, S6), for example Energy, Hunting, Species Introductions (Note: Species Introductions refer to non-native species of any trophic level that may negatively impact the large carnivores via predation, competition, habitat degradation, etc.). Prior to converting threats and following IUCN Red List recommendations, we calculated the ‘impact score’ of each threat-by-species combination, using the ‘severity’, ‘scope’, and ‘timing’ values listed, and removed threats with low impact score. We further removed threats whose timing was listed as ‘future’ as these threats are unlikely to have altered previously recovery outcomes. To test the sensitivity of these restrictions, we compared significant associations with and without low-impact and future threats. The qualitative results were identical with only *Conflict*, *Ecosystem Modification*, and *Hydrological changes* significantly associated with multiple metrics of decline. We then employed binary logistic regression, where negative outcomes were restricted to elevated extinction risk (i.e., threatened status), decreasing trend, and declined status. The estimated change in the odds of observing a particular response associated with each threat or conservation action was based on the coefficients of best-performing models (log odds-ratios). Final models were chosen using forward and backward selection with likelihood ratio testing for the addition or elimination of variables. We explored the use of ordinal logistic regression for ordered, three-category responses (e.g. trend: decreasing → stable → increasing) but model validation showed a violation of the equal proportions assumption. Therefore, we compressed the responses into two categories and employed binary logistic regression. Due to the high proportion of vultures among species showing ongoing declines, we repeated the threats analysis with and without this group to identify associations between negative outcomes and threats that were attributable to vultures alone.

## Supplementary Information

Supplementary Information.

## Data Availability

All relevant data will be made available at https://github.com/ingemanKE/large-carnivore-recoveries/releases/tag/publication_v1.0.0.

## Code availability

All relevant code will be made available at https://github.com/ingemanKE/large-carnivore-recoveries/releases/tag/publication_v1.0.0.
