# Supplementary material for: Glimmers of hope in large carnivore recoveries
Source: Sci Rep. 2022 Jul 21;12:10005. doi: 10.1038/s41598-022-13671-7 (PMC9304400; doi:10.1038/s41598-022-13671-7)
Supplement: Supplementary file 1 — Supplementary Information. [file 41598_2022_13671_MOESM1_ESM.docx]

Supporting Information

Figures S1-S4

Fig S1


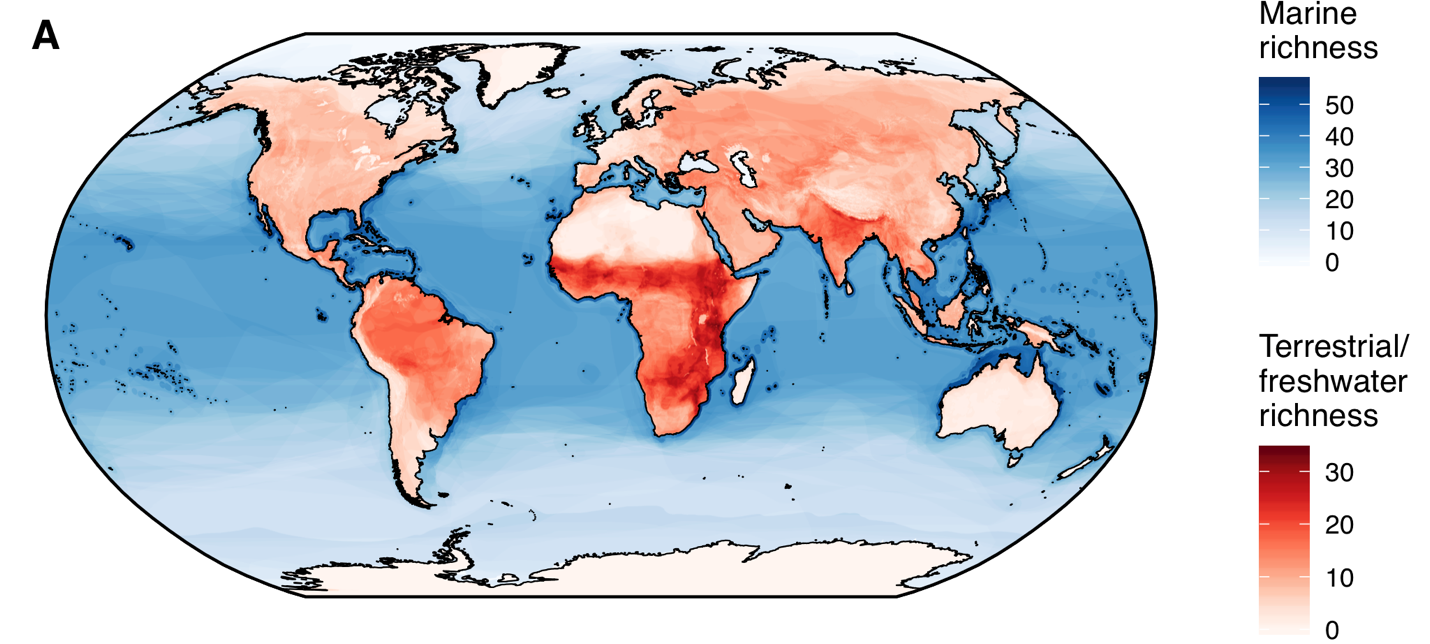


**Figure S1 | Apex Predator Species Richness**. Species ranges from IUCN Red List and BirdLife International are overlaid and summed to produce values of apex predator richness in each 5 km grid. Terrestrial and freshwater species are combined for visual purposes (but not in other analyses). Separate scales are used for marine and terrestrial/freshwater.

**Fig S2**

**
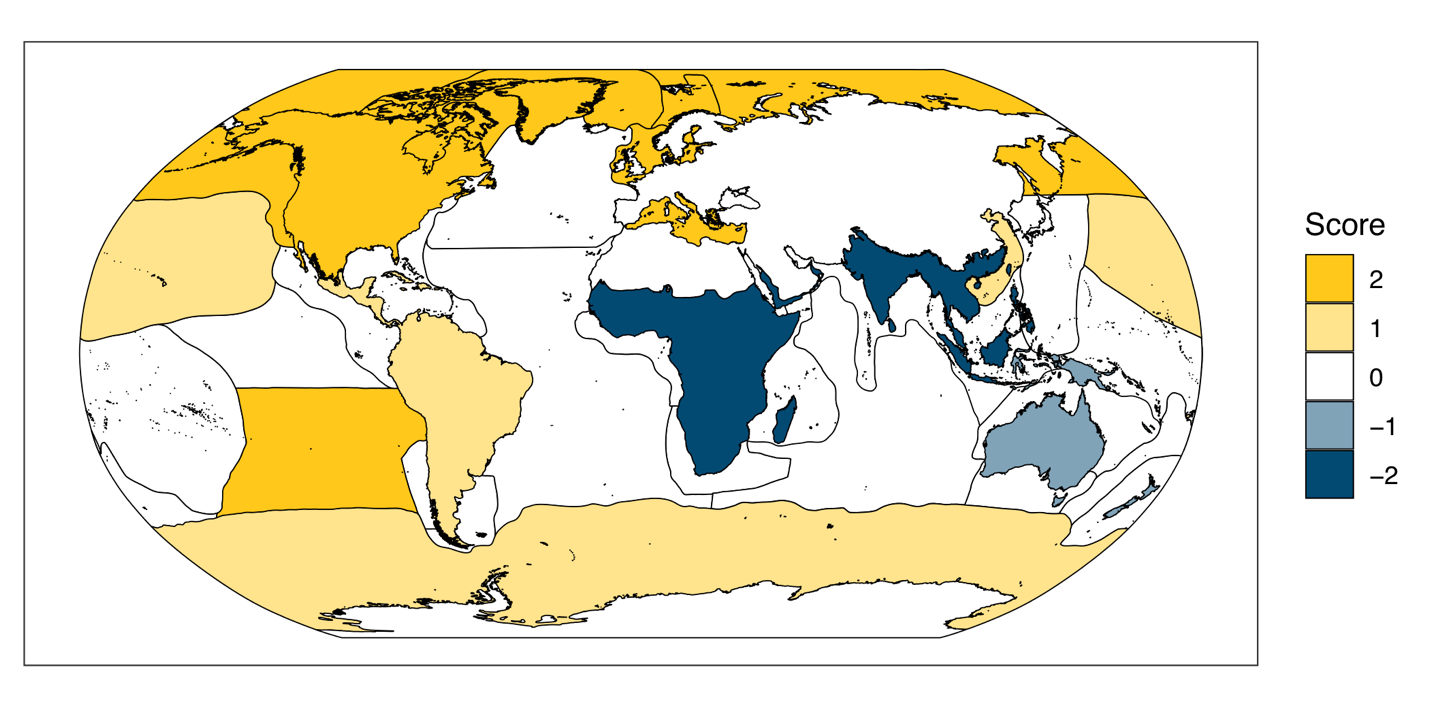
**

**Figure S2 | Biogeographic Realms and Recovery Outcomes** Outlines of the terrestrial/freshwater and marine biogeographic realms used for analysis. Here, realms are scored based on whether recoveries were more or less common within the realm compared with a null expectation proportional to the frequency of recovery across the total data set. A score of *2* indicates that both status improvements and population increases were significantly more common than expected, while *-2* indicates that observations for both of these same recovery metrics were disproportionately lower than expected in that realm. Cases where recovery metrics yielded opposing metrics (scored 0) are not distinguished from cases with no significant deviations from expectations (also scored 0).

**Fig S3**

**
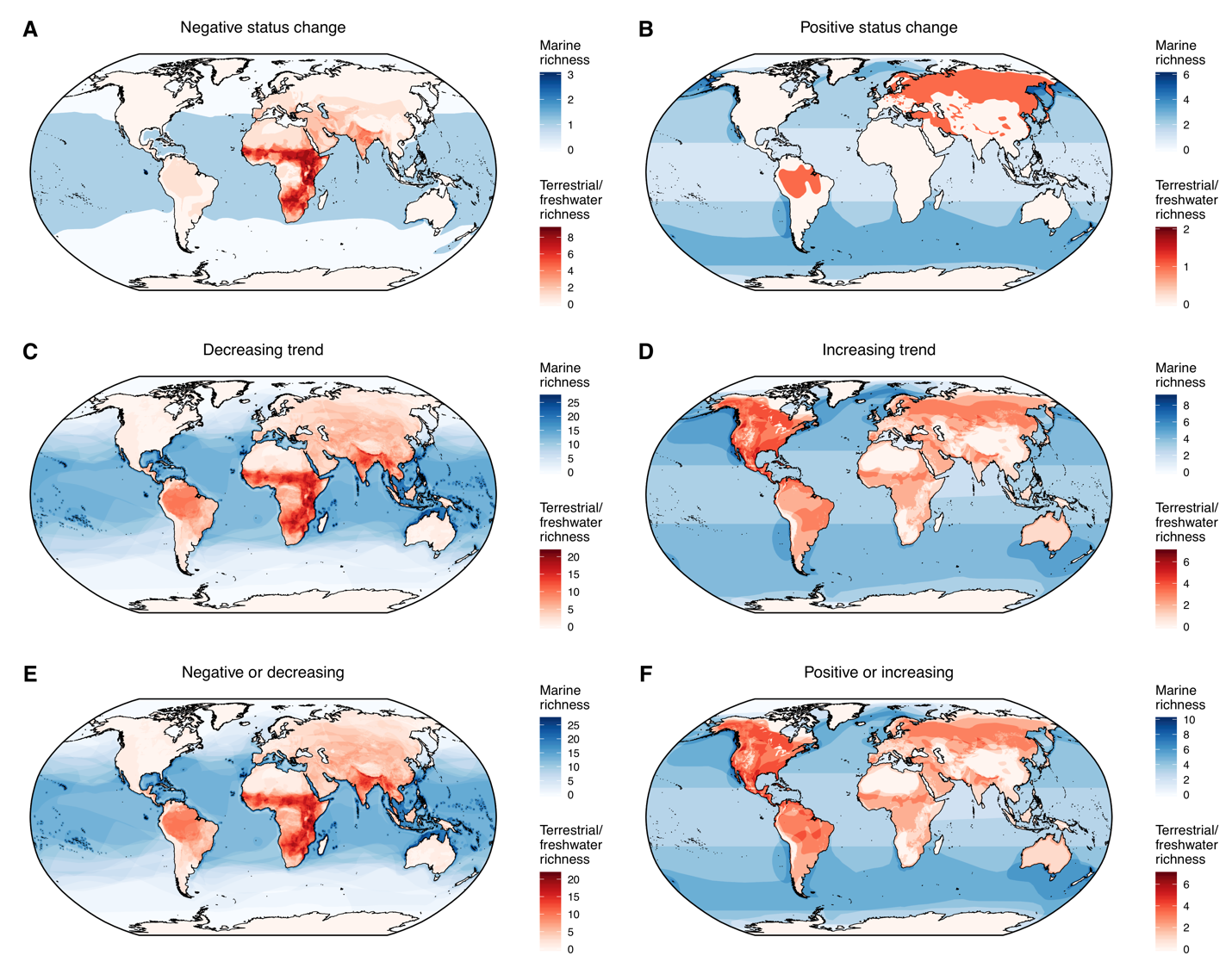
**

**Figure S3 | Positive and Negative Outcomes**. Similarly, to S1, the number of species each species in each 5 km grid was summed to obtain a richness value. Here, however, maps are restricted to species with positive or negative outcomes. **A,** Decline in status since initial assessment. **B,** An improvement in status since initial assessment. **C**, Decreasing population trend. **D,** Increasing population trend. **E** and **F,** Species richness with the two metrics combined.

**Fig S4**

**Figure S4 | Age of IUCN Assessment**. **A,** Median age of assessment across vertebrate classes differ significantly (non-parametric Kruskal-Wallis Test, chi-squared = 161, df = 5, p < 0.0001). Letters represent statistically significant differences between groups from post-hoc tests with alpha = 0.001 (Dunn and Mann-Whitney Tests). **B,** Cumulative frequency distribution for assessment dates by vertebrate class. **C,** Histogram of assessment dates by vertebrate class.

**Tables S1 – S9**

**Table S1: Extinction Risk (Status) by Major Vertebrate Group**

| **Taxon** | **Status** | **Percentage (%)** |
| --- | --- | --- |
| Sharks and Rays | LC | 17.4 |
|  | NT | 21.7 |
|  | VU | 34.8 |
|  | EN | 15.2 |
|  | CR | 10.9 |
| Bony Fishes | LC | 56.2 |
|  | NT | 8.2 |
|  | VU | 15.1 |
|  | EN | 9.6 |
|  | CR | 11.0 |
| Reptiles | LC | 53.8 |
|  | NT | 5.1 |
|  | VU | 17.9 |
|  | EN | 2.6 |
|  | CR | 20.5 |
| Birds | LC | 52.7 |
|  | NT | 14.5 |
|  | VU | 15.5 |
|  | EN | 6.4 |
|  | CR | 10.9 |
| Land Mammals | LC | 30.8 |
|  | NT | 19.2 |
|  | VU | 19.2 |
|  | EN | 26.9 |
|  | CR | 3.8 |
| Marine Mammals | LC | 69.1 |
|  | NT | 4.4 |
|  | VU | 8.8 |
|  | EN | 16.2 |
|  | CR | 1.5 |

Table S2: Prevalence of Recoveries and Ongoing Declines by Major Vertebrate Taxa

| **POPULATION TREND**  Fisher’s exact test p > 0.001 | | | | | | **CHANGE-IN-STATUS**  Fisher’s exact test p = 0.003 | | | | |
| --- | --- | --- | --- | --- | --- | --- | --- | --- | --- | --- |
|  | Chi-square Goodness-of-fit | Post-hoc binomial tests  (Expected / Observed)  p-value | | | Outcome (Qualitative) | Chi-square Goodness-of-fit | Post-hoc binomial tests  (Expected/Observed)  p-value | | | Outcome (Qualitative) |
| **TAXON** |  | **Decreasing** | **Stable** | **Increasing** |  |  | **Declined** | **Unchanged** | **Improved** |  |
| Bony Fishes  – / – | **0.001** | (16 / 29)  **< 0.001** | (24 / 10)  **< 0.001** | (< 1 / 2) 0.32 | **Negative** | **< 0.001** | (< 1 / 4)  **< 0.001** | (75 / 71)  **< 0.001** | (< 1 / 0) 1.0 | **Negative** |
| Sharks  – / 0 | **0.040** | (27 / 21)  **0.047** | (8 / 3)  0.11 | (< 1 / 0)  1.0 | **Negative** | * | (0 / 0) | (47 / 46) | (0 / 1) | **–** |
| Reptiles  – / – | **0.002** | (7 / 12)  0.12 | (19 / 12)  **0.020** | (< 1 / 2)  0.077 | **Negative** | **< 0.001** | (< 1 / 2)  **0.002** | (39 / 36)  **0.001** | (< 1 / 1)  0.289 | **Negative** |
| Birds  0 / – | 0.276 | (61 / 64) | (37 / 30) | (11 / 14) | **–** | **< 0.001** | (2 / 12)  **< 0.001** | (108 / 97)  **< 0.001** | (< 1 / 1) 1.0 | **Negative** |
| Land Mammals  0 / – | 0.391 | (18 / 17) | (4 / 6) | (< 1 / 0) | **–** | 0.474 | (< 1 / 1) | (24 / 24) | (< 1 / 0) | **–** |
| Marine Mammals  + / + | **< 0.001** | (18 / 12)  0.153 | (8 / 4)  0.447 | (7 / 16)  **< 0.001** | **Positive** | **< 0.001** | (1 / 4) 0.081 | (64 / 56)  **0.006** | (2 / 8)  **< 0.001** | **Positive** |

* Expected values of zero cause p-values to be unreliable. However, distribution of observed outcomes does not differ from null expectations.

**Table S2: Prevalence of Recoveries and Ongoing Declines by Major Vertebrate Taxa.** We compared the distribution of recovery outcomes (population trend and change-in-status) for each biogeographical realm to the distribution of outcomes for the broader IUCN Red List. Among terrestrial realms, the Nearctic showed a higher than expected proportion of both recovery metrics, while the Indo-Malay realm showed disproportionate declines. Among marine realms, only the Southern Ocean had higher than expected values for both indicators of recovery. Several temperate realms showed positive indications in at least one of the recovery metrics**.**

**Table S3: Prevalence of Recoveries and Ongoing Declines by Biogeographic Realm**

| **POPULATION TREND**  Fisher’s exact test p > 0.001 | | | | | | **CHANGE-IN-STATUS**  Fisher’s exact test p = 0.003 | | | | |
| --- | --- | --- | --- | --- | --- | --- | --- | --- | --- | --- |
|  | Chi-square Goodness-of-fit | Post-hoc binomial tests  (Expected / Observed)  p-value | | | Outcome (Qualitative) | Chi-square Goodness-of-fit | Post-hoc binomial tests  (Expected/Observed)  p-value | | | Outcome (Qualitative) |
| **Biogeographic**  **Realm** |  | **Decreasing** | **Stable** | **Increasing** |  |  | **Declined** | **Unchanged** | **Improved** |  |
| **Terrestrial** | | | | | | | | | | |
| Afrotropic  – / – | **0.005** | (26 / 38)  **0.008** | (33 / 19)  **0.001** | (2 / 4) 0.480 | **Negative** | **0.005** | (< 1 / 11)  **< 0.001** | (69 / 59)  **< 0.001** | (< 1 / 0) 1.0 | **Negative** |
| Antarctic  0 / 0 | 0.878 | (1 / 1) | (2 / 2) | (< 1 / 0) | **­–** | .965 | (<1 / 0) | (7 / 7) | (<1 / 0) | **–** |
| Australasia  * / 0 | **0.003** | (4 / 6)  0.55 | (5 / 0)  **0.003** | (< 1 / 3)  **0.009** | Mixed^†^ | **0.030** | (< 1 / 2)  0.072 | (34 / 32)  .14 | (< 1 / 0)  1.0 | **–** |
| Indo-Malay  – / – | **< 0.001** | (24 / 41)  **< 0.001** | (31 / 12)  **< 0.001** | (2 / 4)  .403 | **Negative** | **0.005** | (<1 / 5)  **< 0.001** | (64 / 59)  **< 0.001** | (< 1 / 1)  0.55 | **Negative** |
| Nearctic  + / + | **< 0.001** | (14 / 8)  0.146 | (17 / 9)  **0.012** | (1 / 15)  **< 0.001** | **Positive** | **< 0.001** | (< 1 / 1) 0.788 | (43 / 39)  **0.003** | (< 1 / 3)  **0.001** | **Positive** |
| Neotropic  + / * | **< 0.001** | (21 / 20)  1.0 | (27 / 17)  **< 0.001** | (1 / 13)  **< 0.001** | **Positive** | **< 0.001** | (< 1 / 4) **0.002** | (58 / 52)  **0.003** | (< 1 / 3)  **< 0.001** | Mixed |
| Oceania  0 / 0 | **0.027** | (<1 / 1)  1.0 | (1 / 0)  0.l642 | (<1 / 1)  .204 | **–** | **0.985** | (<1 / 0) | (3 / 3) | (<1 / 0) | **–** |
| Palearctic  * / * | **< 0.001** | (33 / 49)  **< 0.001** | (41 / 19)  **< 0.001** | (3 / 9)  **0.004** | Mixed | **< 0.001** | (<1 / 11)  **< 0.001** | (57 / 53)  **< 0.001** | (<1 / 2)  **< 0.001** | Mixed |
| **Biogeographic**  **Realm** |  | **Decreasing** | **Stable** | **Increasing** |  |  | **Declined** | **Unchanged** | **Improved** |  |
| **Marine** | | | | | | | | | | |
| Black Sea | **< 0.001** | (4 / 6)  0.554 | (5 / 0)  **0.003** | (< 1 / 3)  **0.009** | Mixed | **< 0.001** | (< 1 / 1)  0.283 | (14 / 11)  **0.001** | (< 1 / 2)  **0.003** | Mixed |
| Coastal Tropic / Warm Temperate | **< 0.001** | (43 / 67)  **< 0.001** | (54 / 14)  **< 0.001** | (3 / 20)  **< 0.001** | Mixed | **< 0.001** | (<1 / 6)  **< 0.001** | (154 / 140)  **< 0.001** | (< 1 / 10)  **< 0.001** | Mixed |
| Inner Baltic | **< 0.001** | (3 / 2)  1.0 | (3 / 1)  **0.002** | (<1 / 3)  .307 | **Positive** | **0.04** | (<1 / 0)  1.0 | (10 / 9)  0.092 | (<1 / 1)  0.292 | **–** |
| Mid-tropical North Pacific | **< 0.001** | (14 / 24)  **0.002** | (18 / 2)  **< 0.001** | (1 / 7)  **< 0.001** | Mixed | **0.001** | (<1 / 1)  1.0 | (59 / 56)  **0.003** | (<1 / 3)  **0.01** | Mixed |
| Northern Temperate and Arctic Oceans | **< 0.001** | (23 / 35)  **0.007** | (30 / 5)  **< 0.001** | (2 / 15)  **< 0.001** | Mixed | **< 0.001** | (<1 / 1)  1.0 | (95 / 88)  **< 0.001** | (<1 / 7)  **< 0.001** | **Positive** |
| Northwest Pacific | **< 0.001** | (18 / 32)  **< 0.001** | (22 / 3)  **< 0.001** | (1 / 6)  **0.008** | Mixed | **< 0.001** | (<1 / 1)  1.0 | (72 / 68)  **0.002** | (<1 / 4)  **< 0.001** | **Positive** |
| Southeast  Pacific | **< 0.001** | (14 / 23)  **0.003** | (17 / 2)  **< 0.001** | (1 / 7)  **< 0.001** | Mixed | **< 0.001** | (<1 / 1)  1.0 | (57 / 53)  **< 0.001** | (<1 / 4)  **< 0.001** | **Positive** |
| Southern Ocean | **< 0.001** | (<1 / 1)  1.0 | (11 / 3)  **< 0.001** | (<1/ 7)  **< 0.001** | **Positive** | **< 0.001** | (<1 / 4)  0.692 | (37 / 32)  **< 0.001** | (<1 / 4)  **< 0.001** | **Positive** |

* Expected values of zero cause p-values to be unreliable. However, distribution of observed outcomes does not differ from null expectations.

† “Mixed” qualitative outcome indicates that both positive and negative outcomes were more common than expected (e.g., more species increasing and more species decreasing compared to null expectations.

**Table S3: Prevalence of Recoveries and Ongoing Declines by Biogeographic Realm**. We compared the distribution of recovery outcomes for each apex predator taxon to the distribution of outcomes from the larger IUCN Red List for that same taxon. Apex bony fishes and sharks are decreasing at higher frequency than expected. Bony fishes, reptiles, and birds have experienced more status declines than expected. In contrast, marine mammals have larger proportions of increasing and improved species than expected, based on marine mammal outcomes from the broader Red List.

**Table S4: Species experiencing recoveries and ongoing declines**

| **Glimmers of Hope** | | | |
| --- | --- | --- | --- |
| **Scientific** | **Common** | **Status** | **Trend** |
| **Arctocephalus philippii** | **Juan Fernández Fur Seal** | **LC** | **Increasing** |
| **Arctocephalus townsendi** | **Guadalupe Fur Seal** | **LC** | **Increasing** |
| **Balaena mysticetus** | **Bowhead Whale** | **LC** | **Increasing** |
| **Balaenoptera physalus** | **Fin Whale** | **VU** | **Increasing** |
| **Dipturus innominatus** | **Smooth Skate** | **LC** | **Stable** |
| **Eschrichtius robustus** | **Gray Whale** | **LC** | **Stable** |
| **Eumetopias jubatus** | **Steller Sea Lion** | **NT** | **Increasing** |
| **Haliaeetus albicilla** | **White-tailed Sea-eagle** | **LC** | **Increasing** |
| **Lynx pardinus** | **Iberian lynx** | **EN** | **Increasing** |
| **Megaptera novaeangliae** | **Humpback Whale** | **LC** | **Increasing** |
| Melanosuchus niger | Black Caiman | LR/cd | Unknown |
| Aquila adalberti | Spanish Imperial Eagle | VU | Increasing |
| Aquila audax | Wedge-tailed Eagle | LC | Increasing |
| Arctocephalus australis | South American Fur Seal | LC | Increasing |
| Arctocephalus forsteri | New Zealand Fur Seal | LC | Increasing |
| Arctocephalus pusillus | Afro-Australian Fur Seal | LC | Increasing |
| Balaenoptera borealis | Sei Whale | EN | Increasing |
| Balaenoptera musculus | Blue Whale | EN | Increasing |
| Buteo jamaicensis | Red-tailed Hawk | LC | Increasing |
| Buteo regalis | Ferruginous Hawk | LC | Increasing |
| Caracara cheriway | Crested Caracara | LC | Increasing |
| Caracara plancus | Southern Caracara | LC | Increasing |
| Conger conger | Conger Eel | LC | Increasing |
| Coragyps atratus | American Black Vulture | LC | Increasing |
| Crocodylus acutus | American Crocodile | VU | Increasing |
| Gavialis gangeticus | Gharial | CR | Increasing |
| Gymnogyps californianus | California Condor | CR | Increasing |
| Gyps fulvus | Griffon Vulture | LC | Increasing |
| Haliaeetus leucocephalus | Bald Eagle | LC | Increasing |
| Halichoerus grypus | Grey Seal | LC | Increasing |
| Ictalurus punctatus | Channel Catfish | LC | Increasing |
| Lophaetus occipitalis | Long-crested Eagle | LC | Increasing |
| Mirounga angustirostris | Northern Elephant Seal | LC | Increasing |
| Monachus monachus | Mediterranean Monk Seal | EN | Increasing |
| Pagophilus groenlandicus | Harp Seal | LC | Increasing |
| Pandion haliaetus | Osprey | LC | Increasing |
| Strix nebulosa | Great Grey Owl | LC | Increasing |
| Zalophus californianus | Californian Sea Lion | LC | Increasing |
| Eubalaena australis | Southern Right Whale | LC | Unknown |
| **Species in Peril** | | | |
| Aquila nipalensis | Steppe Eagle | EN | Decreasing |
| Arctocephalus galapagoensis | Galapagos Fur Seal | EN | Decreasing |
| Crocodylus rhombifer | Cuban Crocodile | CR | Unknown |
| Gavialis gangeticus | Gharial | CR | Increasing |
| Gyps africanus | White-backed Vulture | CR | Decreasing |
| Gyps coprotheres | Cape Vulture | EN | Decreasing |
| Gyps rueppelli | Rüppell's Vulture | CR | Decreasing |
| Huso dauricus | Kaluga | CR | Decreasing |
| Huso huso | Beluga | CR | Decreasing |
| Hyporthodus ergastularius | Sevenbar Grouper | NT | Decreasing |
| Milvus milvus | Red Kite | NT | Decreasing |
| Necrosyrtes monachus | Hooded Vulture | CR | Decreasing |
| Neophoca cinerea | Australian Sea Lion | EN | Decreasing |
| Neophron percnopterus | Egyptian Vulture | EN | Decreasing |
| Panthera pardus | Leopard | VU | Decreasing |
| Phocarctos hookeri | New Zealand Sea Lion | EN | Decreasing |
| Polemaetus bellicosus | Martial Eagle | EN | Decreasing |
| Sarcogyps calvus | Red-headed Vulture | CR | Decreasing |
| Spizaetus ornatus | Ornate Hawk-eagle | NT | Decreasing |
| Thunnus albacares | Yellowfin Tuna | NT | Decreasing |
| Torgos tracheliotos | Lappet-faced Vulture | EN | Decreasing |
| Trigonoceps occipitalis | White-headed Vulture | CR | Decreasing |
| Zalophus wollebaeki | Galápagos Sea Lion | EN | Decreasing |
|  |  |  |  |
| Glimmers of Hope species in **boldface** have improved status.  Species in Peril in this table include only species with status decline. See Table SX for all species with decreasing population trends. | | | |

**Table S5: IUCN-defined and Author-defined Conservation Actions**

| **IUCN-defined Conservation Actions In-Place** | | | | |
| --- | --- | --- | --- | --- |
| **Action** | **Category** | | **Prompt / Guidance** | |
| Recovery Plan | Monitoring and Planning | | Is there an Action Recovery Plan?  Is there a Recovery Plan, Species Conservation Plan, etc. in place? Select "Yes", "No" or "Unknown". | |
| Monitoring | Monitoring and Planning | | Is there a systematic monitoring scheme?  Is there a monitoring scheme in place specifically for the taxon being assessed? Select "Yes", "No" or "Unknown". Such schemes include: regular census counts, periodic aerial transects, fixed-point photographs, etc. | |
| Conservation Site ID | Land/Water Protection and Management | | Have conservation sites been identified?  Select "Yes, over entire range", "Yes, over part of range", "No" or "Unknown". Note that these should be sites which may or may not be currently receiving any protection, but they have been identified as being important areas e.g., Key Biodiversity Areas, Important Bird Areas, Important Plant Areas, Ecologically and Biologically Significant Areas (marine), etc. | |
| Area Protection | Land/Water Protection and Management | | Does the taxon occur in at least one Protected Area?  Select "Yes", "No" or "Unknown". Protected Areas are usually those that fall within the IUCN Protected Area Categories of I - VI. | |
| Management Plan | Land/Water Protection and Management | | Is there an area based regional management plan?  Is there a Management Plan in place for the Protected Areas or Conservation Sites identified above (this also includes area management plans for parts of the species range outside of protected areas/conservation sites)? Select "Yes", "No" or "Unknown". | |
| Harvest Plan | Species Management | | Is there a harvest management plan?  Select "Yes", "No" or "Unknown". | |
| Ex-situ Management | Species Management | | Is the taxon subject to ex-situ conservation?  Select "Yes", "No" or "Unknown". If yes, details of the ex-situ actions should be provided in the Conservation Actions narrative field. | |
| Education and Awareness | Education and Legislation | | Is the taxon the subject of any recent education or awareness programs?  Select "Yes", "No" or "Unknown". If yes, provide details in the Conservation Actions narrative field. | |
| International Trade | Education and Legislation | | Is the taxon subject to any international management/trade controls?  Select "Yes", "No" or "Unknown". If yes, indicate the Convention, Treaty or Organization involved under the Conservation Actions narrative field if it has not already been specified. *Examples include CITES, Regional Fisheries Agreements, ICCAT, Marine Stewardship Council, Forestry Stewardship Council, Marine Aquarium Council, Phytosanitary Measures Agreement, US Endangered Species Act, etc.* | |
| International legislation | Education and Legislation | | Is the taxon included in international legislation?  Select "Yes", "No" or "Unknown". If "Yes", specify in both the text box and the Conservation Actions narrative which legislation, e.g., the CITES Appendices (indicate which Appendix), CMS Appendices, any of the CMS Regional Agreements (ASCOBAMS, AEWA, ACCOBAMS, EUROBATS, ACAP, etc.), Bern Convention, European Habitats Directive, International Whaling Convention, ICCAT, etc. | |
| **Author-defined Conservation Actions** | | | | |
| **Narrow Action** | | **Category** | | **Description** |
| Harvest | | Wildlife | | Harvest management to ensure sustainable mortality |
| Accidental | | Wildlife | | Reduction of accidental mortality (autos, by-catch, entanglements) |
| Captive breeding | | Reproductive | | Reproductive enhancement in zoos or otherwise |
| Reintroduction | | Reproductive | | Reintroduction or relocation into formerly occupied habitats |
| Habitat restoration | | Reproductive | | Active restoration (as opposed to passive protection) |
| Area protection | | Resources | | Parks, refuges, MPAs, etc. |
| Prey | | Resources | | Prey protection or provisioning |
| Education | | Human | | Education and awareness programs |
| Conflict mitigation | | Human | | Reducing human wildlife conflict |
| Livelihood | | Human | | Livelihood support to reduce poaching |
| National | | Legal | | Species legislation at the national level (ESA, MMPA) |
| International | | Legal | | International agreements (IWC moratorium) |
| Trade | | Legal | | Trade restrictions such as CITES |
| Relocation | | Reproductive | | Outbreeding of genetically isolated populations to reduce inbreeding |

**Table S5.** IUCN-defined and author-defined conservation actions in-place. IUCN Red List Conservation Actions In-place categories and descriptions are adapted from guidance at: <https://www.iucnredlist.org/resources/conservation-actions-classification-scheme>. The authors supplemented analysis of these conservation actions by developing 15 categories of conservation action (nested within 5 broad categories) and tested whether any were associated with increased odds of *increasing* trend or *improved status* using binomial logistic regression.

| **Threat** | **Description** |
| --- | --- |
| Agriculture | Terrestrial farming and cultivation of perennial and annual crops (excluding timber) |
| Aquaculture | Cultivation of aquatic marine and freshwater species |
| Bycatch | Mortality and loss due to the unintended capture by fisheries |
| Climate change | Direct and indirect effects of changing environmental conditions and associated severe weather |
| Conflict | War, unrest, and instability due to human conflict |
| Development | Urbanization and expansion of residential, commercial, and industrial areas |
| Disease | Mortality and reproductive decline due to known or unknown diseases |
| Ecosystem modification | Degradation, conversion, modification, or loss of ecosystems that represent habitat for focal species |
| Energy | Development and operation of oil and gas, mining, or renewable energy sources |
| Fire | Fire (controlled and uncontrolled) and fire suppression |
| Fishing | Legal exploitation of marine and freshwater species by industrial, commercial, or recreational; sea-going or shore-based fishing |
| Geological | Volcanoes, earthquakes, avalanches, tsunamis and related geological events |
| Human intrusion | Disturbance by encroachment of human activities (excluding existing categories such as recreation, energy production, etc.) |
| Hunting | Legal exploitation of terrestrial species for subsistence or medicinal purposes |
| Hydrological modification | Alteration of hydrological properties of ecosystems by the development of dams or the abstraction of ground or surface water |
| Livestock | Direct and indirect effects of livestock farming, ranching, and grazing by nomadic, small-holder, and industrial-scale actors |
| Other threats | Threats that due not fall under existing categories (excluded from analysis) |
| Persecution | Direct killing, culling, or hazing for the purpose of population control or to reduce conflict with human livelihoods |
| Pollution | Effects of sewage, refuse and waste; nutrients, effluents, runoff, and spills; acid rain and smog, thermal, light, or noise pollution. |
| Recreation | Disturbance, injury, or mortality as a result of incursion by human recreational activities (e.g., propellor injuries due to recreational boating) |
| Species introduction | Direct or indirect effects of non-native, introduced, or invasive species through modification of habitat, predation, competition or other |
| Timber | Extraction and processing of timber resources, including legal and illegal logging, including subsistence, small-scale, large-scale, and industrial operations |
| Transportation | Disturbance, injury, or mortality due to the development or operation of roads, railroads, shipping lines, flight paths, etc. |

**Table S6: Recategorized Threats**

**Table S6.** Description of author-defined, recategorized threats representing the relevant sector or human activity threatening the focal species.

**Table S7: Mapping of Threats to IUCN Threat and Stress codes**

| **Threat** | **IUCN Codes** | | **Threat** | **IUCN Codes** | | **Threat** | **IUCN Codes** | |
| --- | --- | --- | --- | --- | --- | --- | --- | --- |
| Agriculture | 2.1.1 1.1  2.1.1 1.2  2.1.1 2.1  2.1.1 2.2  2.1.2 1.1  2.1.2 1.2  2.1.2 2.1  2.1.2 2.2  2.1.2 2.3.7  2.1.2 2.3.8  2.1.3 1.1  2.1.3 1.2  2.1.3 2.1  2.1.3 2.2  2.1.3 2.3.7  2.1.3 2.3.8  2.1.4 1.1  2.1.4 1.2  2.1.4 2.2  9.3.1 1.2 | 9.3.1 1.3 9.3.1 2.2  9.3.2 1.1  9.3.2 1.2  9.3.2 1.3  9.3.2 2.1  9.3.2 2.2  9.3.2 2.3.7  9.3.3  9.3.3 1.1  9.3.3 1.2  9.3.3 1.3  9.3.3 2.1  9.3.3 2.2  9.3.3 2.3.7  9.3.3 2.3.8  9.3.4  9.3.4 1.2  9.3.4 2.1  9.3.4 2.3.7 | Energy | 3.1  3.2  3.1 1.1  3.1 1.2  3.1 2.1  3.1 2.2  3.1 2.3.7  3.2 1.1  3.2 1.2  3.2 1.3  3.2 2.1  3.2 2.2  3.2 2.3.7  3.3 1.1 | 3.3 1.2  3.3 2.1  3.3 2.2  9.2.1  9.2.1 1.1  9.2.1 1.2  9.2.1 2.1  9.2.1 2.2  9.2.1 2.3.7  9.2.2  9.2.2 1.1  9.2.2 1.2  9.2.2 2.2 | Persecution | 5.4.5  5.4.5 1.3  5.4.5 2.1 | 5.4.5 2.2  5.4.6 1.2  5.4.6 2.1 |
| Aquaculture | 2.4.2 1.1  2.4.2 2.3.1 | 2.4.3 1.1  2.4.3 1.2 | Fire | 7.1.1 1.1  7.1.1 1.2  7.1.1 2.1  7.1.2 1.1 | 7.1.2 1.2  7.1.2 2.1  7.1.3 1.2 | Pollution | 9.2.3  9.2.3 1.2  9.2.3 2.1  9.2.3 2.3.8  9.4 1.1  9.4 1.2 | 9.4 2.1  9.4 2.3.7  9.5.4 1.2  9.5.4 2.3.8  9.6.3 |
| Bycatch | 5.4.3  5.4.3 2.1  5.4.3 2.2  5.4.3 2.3.8  5.4.4 | 5.4.4 2.1  5.4.4 2.2  5.4.4 2.3.7  5.4.4 2.3.8 | Fishing | 5.4.1  5.4.1 1.2  5.4.1 1.3  5.4.1 2.1  5.4.1 2.2  5.4.1 2.3.7  5.4.2  5.4.2 1.2  5.4.2 1.3 | 5.4.2 2.1  5.4.2 2.2  5.4.2 2.3.7  5.4.3 1.2  5.4.3 1.3  5.4.4 1.2  5.4.4 1.3  5.4.4 2.3.2 | Recreation | 6.1  1.3 1.1  1.3 1.2  1.3 2.2  6.1 1.2  6.1 1.3  6.1 2.1  6.1 2.2  6.1 2.3.7 |  |
| Climate change | 11.1  11.2  11.3  11.4  11.1 1.1  11.1 1.2  11.1 1.3  11.1 2.1  11.1 2.2  11.1 2.3.2  11.1 2.3.7  11.1 2.3.8  11.2 1.1  11.2 1.2  11.2 2.1  11.2 2.2 | 11.3 1.1  11.3 1.2  11.3 1.3  11.3 2.1  11.3 2.2  11.3 2.3.7  11.3 2.3.8  11.4 1.2  11.4 2.1  11.4 2.2  11.5 1.1  11.5 1.2  11.5 1.3  11.5 2.1  11.5 2.2  11.5 2.3.7 | Hunting | 5.1.1 1.1  5.1.1 1.2  5.1.1 2.1  5.1.1 2.2  5.1.1 2.3.7  5.1.2 1.2  5.1.2 1.3  5.1.2 2.1  5.1.2 2.2 | 5.1.2 2.3.2  5.1.2 2.3.7  5.1.2 2.3.8  5.1.3 1.2  5.1.3 2.1  5.1.3 2.2  5.1.3 2.3.7  5.2.2 1.2  5.2.2 1.3 | Species introduction | 8.1.1  8.1.1 1.2  8.1.1 2.1  8.1.1 2.2  8.1.1 2.3.1  8.1.1 2.3.2  8.1.1 2.3.7  8.1.2  8.1.2 1.2 | 8.1.2 1.3  8.1.2 2.1  8.1.2 2.2  8.1.2 2.3.1  8.1.2 2.3.2  8.1.2 2.3.7  8.1.2 2.3.8  8.3 2.3.1 |
| Conflict | 6.2 1.2  6.2 2.1 | 6.2 2.2 | Geological | 10.2 1.1 |  | Other Threats | 12.1 1.2  12.1 2.1 | 12.1 2.3.7  12.1 2.3.8 |
| Development | 1.2  1.1 1.1  1.1 1.2  1.1 2.1  1.1 2.2  1.1 2.3.7  1.2 1.1  1.2 1.2  1.2 2.2  9.1.1  9.1.1 1.1  9.1.1 1.2  9.1.1 2.1 | 9.1.1 2.2  9.1.2  9.1.2 1.2  9.1.2 2.1  9.1.2 2.2  9.1.3  9.1.3 1.2  9.1.3 2.2  9.6.3 1.2  9.6.3 2.1  9.6.3 2.2  9.6.3 2.3.7 | Human Intrusion | 6.3 1.2  6.3 2.1 | 6.3 2.2  6.3 2.3.7 | Timber | 2.2.1 1.1  2.2.1 1.2  2.2.2 1.1  2.2.2 1.2  2.2.2 2.1  2.2.2 2.2  2.2.2 2.3.7  2.2.3 1.1  2.2.3 1.2  5.1.3  5.3.1 1.1  5.3.1 1.2 | 5.3.1 1.2  5.3.1 2.2  5.3.2 1.1  5.3.2 1.2  5.3.3  5.3.3 1.1  5.3.3 1.2  5.3.3 2.3.7  5.3.4 1.1  5.3.4 1.2  5.3.4 2.2  5.3.5 1.2 |
| Disease | 8.2  8.6  8.2 1.2  8.2 2.1  8.2 2.2  8.2 2.3.1  8.2 2.3.2  8.2 2.3.7  8.2.1 1.2  8.2.1 2.1  8.2.1 2.2  8.2.1 2.3.7  8.2.2 2.1 | 8.2.2 2.2  8.2.2 2.3.1  8.2.2 2.3.2  8.2.2 2.3.7  8.5.1 2.1  8.5.1 2.3.7  8.5.2  8.5.2 1.2  8.5.2 2.1  8.5.2 2.2  8.5.2 2.3.7  8.6 2.1  8.6 2.3.7 | Hydrological modification | 7.2.1  7.2.10 1.1  7.2.10 1.2  7.2.10 1.3  7.2.10 2.3.8  7.2.11  7.2.11 1.1  7.2.11 1.2  7.2.11 1.3  7.2.11 2.1  7.2.11 2.2 | 7.2.11 2.3.8  7.2.2 1.2  7.2.3 1.1  7.2.3 1.2  7.2.3 1.3  7.2.3 2.3.7  7.2.4 1.2  7.2.7 1.1  7.2.7 1.2  7.2.8 1.2 | Transportation | 4.2 1.1  4.2 1.2  4.2 2.1  4.2 2.2  4.2 2.3.7  4.3 1.1 | 4.3 1.2  4.3 2.1  4.3 2.2  4.3 2.3.7  4.4 2.1  4.4 2.2 |
| Ecosystem modification | 7.3 1.1  7.3 1.2  7.3 1.3  7.3 2.1 | 7.3 2.2  7.3 2.3.2  7.3 2.3.7 | Livestock | 2.3.1 1.1  2.3.1 1.2  2.3.1 2.1  2.3.1 2.2  2.3.1 2.3.2  2.3.2 1.1  2.3.2 1.2  2.3.2 1.3  2.3.2 2.1 | 2.3.2 2.2  2.3.2 2.3.2  2.3.3 1.1  2.3.3 1.2  2.3.3 2.1  2.3.4 1.1  2.3.4 1.2  2.3.4 2.2 |  |  |  |

**Table S7.** Mapping of author-defined, recategorized threats to IUCN-defined codes combining ‘threats’ (first code, up to three levels, left) and ‘stresses’ (second code, up to three, right). For example, the last threat-stress combination code listed for ‘Ecosystem modification’ (7.3 2.3.7) is mapped to IUCB threat 7.3 (‘Natural system modifications, other ecosystem modifications’) and stress code 2.3.7 (‘Species stresses, reduced reproductive success’). IUCN ‘threat’ and ‘stress’ guidance can be found obtained at <https://www.iucnredlist.org/resources/threat-classification-scheme> and <https://www.iucnredlist.org/resources/stresses-classification-scheme>

**Table S8: Associations between threats and negative outcomes without vultures**

|  | **Association between threats and negative conservation outcomes**  (Vultures excluded) | | | | | |
| --- | --- | --- | --- | --- | --- | --- |
|  | Odds ratio  (95% CI) | P-value  (Chi-square test) | Odds ratio (95% CI) | P-value  (Chi-square test) | Odds ratio (95% CI) | P-value  (Chi-square test) |
| **Threat Category** | **Elevated extinction risk** | | **Decline in status** | | **Decreasing trend** | |
| **Conflict** | **28.3 (3.0 - 1109)** | **0.019** |  |  | 36.8 (3.9 - 1057) | 0.0078 |
| Ecosystem modification |  |  |  |  |  | NS |
| **Species introduction** | **2.8 (1.1 – 7.0)** | **0.029** |  |  |  |  |
| **Human intrusion** | **5.7 (1.5 – 28.5)** | **0.017** |  |  |  |  |
| Persecution |  | NS |  | NS |  |  |
| Aquaculture |  |  |  |  |  |  |
| Transportation |  | NS |  |  |  |  |
| Recreation |  |  |  |  |  |  |
| Hunting |  | NS |  |  |  |  |
| Agriculture |  |  |  |  |  |  |
| **Timber** |  |  |  |  | **5.9 (2.3 – 16.7))** | **0.00036** |
| **Fishing** | **2.8 (1.4 – 5.7)** | **0.0046** |  |  | **4.4 (2.0 – 9.9)** | **0.00024** |
| Bycatch |  |  |  |  |  |  |
| **Hydrological modification** |  |  | **3.4 (1.0 – 11.0)** | **0.034** | **5.4 (1.6 – 23.8)** | **0.0143** |
| Development |  |  |  |  |  |  |
| Disease |  |  |  |  | 0.39 (0.16 – 0.90) | 0.0291 |
| **Energy** |  |  | **3.2 (1.0 – 9.9)** | **0.0462** | 0.24 (0.09 – 0.61) | 0.0031 |
| Pollution | 0.34 (0.14 – 0.78) | 0.015 |  |  | 0.26 (0.08 – 0.79) | 0.020 |
| Climate | 0.39 (0.19 – 0.77) | 0.0081 |  |  |  |  |
| Livestock |  |  |  |  |  |  |
| Fire |  | NS |  |  |  |  |

**Table S8: Results of Logistic Regression with Vultures Excluded**. We repeated the logistic regression analysis of the association between threats and negative conservation outcomes with vultures excluded. Negative outcomes included elevated extinction risk (i.e., threatened status), decreasing trend, and declined status. The estimated change in the odds of observing a particular response associated with each threat was based on the coefficients (log odds-ratios) of best-performing models (based on AIC). Final models were chosen using forward and backward selection with likelihood ratio testing for the addition or elimination of variables. P-values represent chi-square test for the significance of individual threats in the best-performing model. Threats highlighted in **bold** were associated with a significant *increase* in the odds of observing at least one of the indicators of ongoing declines. Threats labeled “NS” where included in the best performing model for that negative outcome but had 95% confidence interval that spanned 1 (non-significant change-in-odds). Threats with no listed odds ratio, p-value, or “NS” were not included in the final model for that particular indicator of decline.

**Table S9: Large Carnivore species list**

| Scientific  name | Common  name | Taxon | Ecosystem(s) | IUCN Status | Status Change | Population  trend |
| --- | --- | --- | --- | --- | --- | --- |
| Aaptosyax grypus | Mekong Giant Salmon Carp | Bony fishes | Freshwater | CR | Unchanged | Decreasing |
| Acanthocybium solandri | Wahoo | Bony fishes | Marine | LC | Unchanged | Stable |
| Accipiter henstii | Henst's Goshawk | Birds | Terrestrial | NT | Unchanged | Decreasing |
| Acinonyx jubatus | Cheetah | Terrestrial mammals | Terrestrial | VU | Unchanged | Decreasing |
| Aegypius monachus | Cinereous Vulture | Birds | Terrestrial | NT | Unchanged | Decreasing |
| Aetobatus narinari | Spotted Eagle Ray | Sharks and rays | Marine | NT | Unchanged | Decreasing |
| Alligator mississippiensis | American Alligator | Reptiles | Freshwater, Terrestrial | LC | Unchanged | Unknown |
| Alligator sinensis | Chinese Alligator | Reptiles | Freshwater, Terrestrial | CR | Unchanged | Stable |
| Alopias pelagicus | Pelagic Thresher | Sharks and rays | Marine | EN | Unchanged | Decreasing |
| Alopias superciliosus | Bigeye Thresher Shark | Sharks and rays | Marine | VU | Unchanged | Decreasing |
| Alopias vulpinus | Common Thresher Shark | Sharks and rays | Marine | VU | Unchanged | Decreasing |
| Andrias davidianus | Chinese Giant Salamander | Reptiles | Freshwater | CR | Unchanged | Decreasing |
| Andrias japonicus | Japanese Giant Salamander | Reptiles | Freshwater | NT | Unchanged | Decreasing |
| Anoxypristis cuspidata | Narrow Sawfish | Sharks and rays | Marine | EN | Unchanged | Decreasing |
| Aonyx capensis | African Clawless Otter | Terrestrial mammals | Marine, Freshwater, Terrestrial | NT | Unchanged | Decreasing |
| Aonyx congicus | Congo Clawless Otter | Terrestrial mammals | Freshwater, Terrestrial | NT | Unchanged | Decreasing |
| Apodora papuana | Papuan Olive Python | Reptiles | Terrestrial | LC | Unchanged | Stable |
| Aquila adalberti | Spanish Imperial Eagle | Birds | Freshwater, Terrestrial | VU | Unchanged | Increasing |
| Aquila africana | Cassin's Hawk-eagle | Birds | Freshwater, Terrestrial | LC | Unchanged | Decreasing |
| Aquila audax | Wedge-tailed Eagle | Birds | Freshwater, Terrestrial | LC | Unchanged | Increasing |
| Aquila chrysaetos | Golden Eagle | Birds | Terrestrial | LC | Unchanged | Stable |
| Aquila fasciata | Bonelli's Eagle | Birds | Freshwater, Terrestrial | LC | Unchanged | Decreasing |
| Aquila gurneyi | Gurney's Eagle | Birds | Terrestrial | NT | Unchanged | Decreasing |
| Aquila heliaca | Eastern Imperial Eagle | Birds | Freshwater, Terrestrial | VU | Unchanged | Decreasing |
| Aquila nipalensis | Steppe Eagle | Birds | Terrestrial | EN | Declined | Decreasing |
| Aquila rapax | Tawny Eagle | Birds | Freshwater, Terrestrial | VU | Unchanged | Decreasing |
| Aquila spilogaster | African Hawk-eagle | Birds | Freshwater, Terrestrial | LC | Unchanged | Decreasing |
| Aquila verreauxii | Verreaux's Eagle | Birds | Terrestrial | LC | Unchanged | Stable |
| Arctocephalus australis | South American Fur Seal | Marine Mammals | Marine, Terrestrial | LC | Unchanged | Increasing |
| Arctocephalus forsteri | New Zealand Fur Seal | Marine Mammals | Marine, Terrestrial | LC | Unchanged | Increasing |
| Arctocephalus galapagoensis | Galapagos Fur Seal | Marine Mammals | Marine, Terrestrial | EN | Declined | Decreasing |
| Arctocephalus gazella | Antarctic Fur Seal | Marine Mammals | Marine, Terrestrial | LC | Unchanged | Decreasing |
| Arctocephalus philippii | Juan Fernández Fur Seal | Marine Mammals | Marine, Terrestrial | LC | Improved | Increasing |
| Arctocephalus pusillus | Afro-Australian Fur Seal | Marine Mammals | Marine, Terrestrial | LC | Unchanged | Increasing |
| Arctocephalus townsendi | Guadalupe Fur Seal | Marine Mammals | Marine, Terrestrial | LC | Improved | Increasing |
| Arctocephalus tropicalis | Subantarctic Fur Seal | Marine Mammals | Marine, Terrestrial | LC | Unchanged | Stable |
| Argyrosomus hololepidotus | Madagascar Kob | Bony fishes | Marine | EN | Unchanged | Unknown |
| Argyrosomus regius | Meagre | Bony fishes | Marine | LC | Unchanged | Unknown |
| Bagrus docmak | Sudan Catfish | Bony fishes | Freshwater | LC | Unchanged | Unknown |
| Balaena mysticetus | Bowhead Whale | Marine Mammals | Marine | LC | Improved | Increasing |
| Balaenoptera acutorostrata | Common Minke Whale | Marine Mammals | Marine | LC | Unchanged | Unknown |
| Balaenoptera borealis | Sei Whale | Marine Mammals | Marine | EN | Unchanged | Increasing |
| Balaenoptera musculus | Blue Whale | Marine Mammals | Marine | EN | Unchanged | Increasing |
| Balaenoptera physalus | Fin Whale | Marine Mammals | Marine | VU | Improved | Increasing |
| Benthodesmus tenuis | NA | Bony fishes | Marine | LC | Unchanged | Unknown |
| Brachyplatystoma rousseauxii | Gilded Catfish | Bony fishes | Freshwater | LC | Unchanged | Unknown |
| Bubo ascalaphus | Pharaoh Eagle-owl | Birds | Freshwater, Terrestrial | LC | Unchanged | Stable |
| Bubo bengalensis | Rock Eagle-owl | Birds | Terrestrial | LC | Unchanged | Stable |
| Bubo bubo | Eurasian Eagle-owl | Birds | Terrestrial | LC | Unchanged | Decreasing |
| Bubo capensis | Cape Eagle-owl | Birds | Terrestrial | LC | Unchanged | Stable |
| Bubo lacteus | Verreaux's Eagle-owl | Birds | Freshwater, Terrestrial | LC | Unchanged | Stable |
| Bubo magellanicus | Magellanic Horned Owl | Birds | Terrestrial | LC | Unchanged | Stable |
| Bubo nipalensis | Spot-bellied Eagle-owl | Birds | Terrestrial | LC | Unchanged | Decreasing |
| Bubo scandiacus | Snowy Owl | Birds | Freshwater, Terrestrial | VU | Unchanged | Decreasing |
| Bubo shelleyi | Shelley's Eagle-owl | Birds | Terrestrial | VU | Unchanged | Decreasing |
| Bubo sumatranus | Barred Eagle-owl | Birds | Terrestrial | LC | Unchanged | Stable |
| Bubo virginianus | Great Horned Owl | Birds | Terrestrial | LC | Unchanged | Stable |
| Buteo augur | Augur Buzzard | Birds | Terrestrial | LC | Unchanged | Stable |
| Buteo galapagoensis | Galapagos Hawk | Birds | Marine, Terrestrial | VU | Unchanged | Stable |
| Buteo hemilasius | Upland Buzzard | Birds | Terrestrial | LC | Unchanged | Stable |
| Buteo jamaicensis | Red-tailed Hawk | Birds | Terrestrial | LC | Unchanged | Increasing |
| Buteo regalis | Ferruginous Hawk | Birds | Terrestrial | LC | Unchanged | Increasing |
| Buteo rufinus | Long-legged Buzzard | Birds | Terrestrial | LC | Unchanged | Stable |
| Buteo rufofuscus | Jackal Buzzard | Birds | Terrestrial | LC | Unchanged | Stable |
| Buteo socotraensis | Socotra Buzzard | Birds | Terrestrial | VU | Unchanged | Stable |
| Buteo ventralis | Rufous-tailed Hawk | Birds | Terrestrial | VU | Unchanged | Stable |
| Buteogallus coronatus | Crowned Solitary Eagle | Birds | Terrestrial | EN | Unchanged | Decreasing |
| Buteogallus schistaceus | Slate-colored Hawk | Birds | Terrestrial | LC | Unchanged | Decreasing |
| Buteogallus solitarius | Black Solitary Eagle | Birds | Terrestrial | NT | Unchanged | Decreasing |
| Buteogallus urubitinga | Great Black Hawk | Birds | Terrestrial | LC | Unchanged | Stable |
| Caiman crocodilus | Common Caiman | Reptiles | Freshwater, Terrestrial | LC | Unchanged | Stable |
| Caiman latirostris | Broad-snouted Caiman | Reptiles | Freshwater, Terrestrial | LC | Unchanged | Unknown |
| Caiman yacare | Yacaré | Reptiles | Freshwater, Terrestrial | LC | Unchanged | Unknown |
| Callorhinus ursinus | Northern Fur Seal | Marine Mammals | Marine, Terrestrial | VU | Unchanged | Decreasing |
| Canis lupus | Gray Wolf | Terrestrial mammals | Terrestrial | LC | Unchanged | Stable |
| Canis rufus | Red Wolf | Terrestrial mammals | Terrestrial | CR | Unchanged | Decreasing |
| Canis simensis | Ethiopian Wolf | Terrestrial mammals | Terrestrial | EN | Unchanged | Decreasing |
| Caracal caracal | Caracal | Terrestrial mammals | Terrestrial | LC | Unchanged | Unknown |
| Caracara cheriway | Crested Caracara | Birds | Freshwater, Terrestrial | LC | Unchanged | Increasing |
| Caracara plancus | Southern Caracara | Birds | Freshwater, Terrestrial | LC | Unchanged | Increasing |
| Caranx ignobilis | Giant Trevally | Bony fishes | Marine | LC | Unchanged | Unknown |
| Carcharhinus albimarginatus | Silvertip Shark | Sharks and rays | Marine | VU | Unchanged | Decreasing |
| Carcharhinus brachyurus | Copper Shark | Sharks and rays | Marine, Freshwater | NT | Unchanged | Unknown |
| Carcharhinus brevipinna | Spinner Shark | Sharks and rays | Marine | NT | Unchanged | Unknown |
| Carcharhinus falciformis | Silky Shark | Sharks and rays | Marine | VU | Unchanged | Decreasing |
| Carcharhinus galapagensis | Galapagos Shark | Sharks and rays | Marine | LC | Unchanged | Unknown |
| Carcharhinus leucas | Bull Shark | Sharks and rays | Marine, Freshwater | NT | Unchanged | Unknown |
| Carcharhinus limbatus | Blacktip Shark | Sharks and rays | Marine | NT | Unchanged | Unknown |
| Carcharhinus longimanus | Oceanic Whitetip Shark | Sharks and rays | Marine | CR | Unchanged | Decreasing |
| Carcharhinus obscurus | Dusky Shark | Sharks and rays | Marine | EN | Unchanged | Decreasing |
| Carcharhinus plumbeus | Sandbar Shark | Sharks and rays | Marine | VU | Unchanged | Decreasing |
| Carcharhinus signatus | Night Shark | Sharks and rays | Marine | VU | Unchanged | Decreasing |
| Carcharhinus tilstoni | Australian Blacktip Shark | Sharks and rays | Marine | LC | Unchanged | Stable |
| Carcharias taurus | Sand Tiger Shark | Sharks and rays | Marine | VU | Unchanged | Unknown |
| Carcharodon carcharias | Great White Shark | Sharks and rays | Marine | VU | Unchanged | Unknown |
| Cathartes aura | Turkey Vulture | Birds | Terrestrial | LC | Unchanged | Stable |
| Cathartes burrovianus | Lesser Yellow-headed Vulture | Birds | Freshwater, Terrestrial | LC | Unchanged | Stable |
| Cathartes melambrotus | Greater Yellow-headed Vulture | Birds | Terrestrial | LC | Unchanged | Decreasing |
| Cephalorhynchus eutropia | Chilean Dolphin | Marine Mammals | Marine, Freshwater | NT | Unchanged | Decreasing |
| Channa marulius | Great snakehead | Bony fishes | Freshwater | LC | Unchanged | Unknown |
| Chitala lopis | Giant Featherback | Bony fishes | Freshwater | LC | Unchanged | Unknown |
| Circaetus beaudouini | Beaudouin's Snake-eagle | Birds | Terrestrial | VU | Unchanged | Decreasing |
| Circaetus cinereus | Brown Snake-eagle | Birds | Terrestrial | LC | Unchanged | Decreasing |
| Circaetus fasciolatus | Southern Banded Snake-eagle | Birds | Terrestrial | NT | Unchanged | Decreasing |
| Circaetus gallicus | Short-toed Snake-eagle | Birds | Terrestrial | LC | Unchanged | Stable |
| Circaetus pectoralis | Black-chested Snake-eagle | Birds | Terrestrial | LC | Unchanged | Unknown |
| Clanga clanga | Greater Spotted Eagle | Birds | Freshwater, Terrestrial | VU | Unchanged | Decreasing |
| Clanga hastata | Indian Spotted Eagle | Birds | Terrestrial | VU | Unchanged | Decreasing |
| Clanga pomarina | Lesser Spotted Eagle | Birds | Terrestrial | LC | Unchanged | Stable |
| Conger conger | Conger Eel | Bony fishes | Marine | LC | Unchanged | Increasing |
| Coragyps atratus | American Black Vulture | Birds | Terrestrial | LC | Unchanged | Increasing |
| Crocodylus acutus | American Crocodile | Reptiles | Marine, Freshwater, Terrestrial | VU | Unchanged | Increasing |
| Crocodylus intermedius | Orinoco Crocodile | Reptiles | Freshwater, Terrestrial | CR | Unchanged | Decreasing |
| Crocodylus johnstoni | Australian freshwater Crocodile | Reptiles | Freshwater, Terrestrial | LC | Unchanged | Stable |
| Crocodylus mindorensis | Philippine Crocodile | Reptiles | Freshwater, Terrestrial | CR | Unchanged | Decreasing |
| Crocodylus moreletii | Morelet's Crocodile | Reptiles | Freshwater, Terrestrial | LC | Unchanged | Stable |
| Crocodylus niloticus | Nile Crocodile | Reptiles | Freshwater, Terrestrial | LC | Unchanged | Stable |
| Crocodylus novaeguineae | New Guinea Crocodile | Reptiles | Freshwater, Terrestrial | LC | Unchanged | Unknown |
| Crocodylus palustris | Mugger | Reptiles | Freshwater, Terrestrial | VU | Unchanged | Stable |
| Crocodylus porosus | Salt-water Crocodile | Reptiles | Marine, Freshwater, Terrestrial | LC | Unchanged | Unknown |
| Crocodylus rhombifer | Cuban Crocodile | Reptiles | Freshwater, Terrestrial | CR | Declined | Unknown |
| Crocodylus siamensis | Siamese Crocodile | Reptiles | Freshwater, Terrestrial | CR | Unchanged | Decreasing |
| Crocuta crocuta | Spotted Hyaena | Terrestrial mammals | Terrestrial | LC | Unchanged | Decreasing |
| Cuon alpinus | Dhole | Terrestrial mammals | Terrestrial | EN | Unchanged | Decreasing |
| Cyclura cornuta | Rhinoceros Iguana | Reptiles | Terrestrial | VU | Unchanged | Decreasing |
| Cyclura stejnegeri | Mona Rhinoceros Iguana | Reptiles | Terrestrial | EN | Unchanged | Decreasing |
| Cystophora cristata | Hooded Seal | Marine Mammals | Marine, Terrestrial | VU | Unchanged | Unknown |
| Delphinapterus leucas | Beluga | Marine Mammals | Marine | LC | Unchanged | Unknown |
| Delphinus delphis | Short-beaked Common Dolphin | Marine Mammals | Marine | LC | Unchanged | Unknown |
| Dendroaspis polylepis | Black Mamba | Reptiles | Terrestrial | LC | Unchanged | Stable |
| Dipturus innominatus | Smooth Skate | Sharks and rays | Marine | LC | Improved | Stable |
| Dolichophis jugularis | Large Whip Snake | Reptiles | Terrestrial | LC | Unchanged | Stable |
| Dolichophis schmidti | Schmidt's Whip Snake | Reptiles | Terrestrial | LC | Unchanged | Stable |
| Epinephelus itajara | Atlantic Goliath Grouper | Bony fishes | Marine | VU | Unchanged | Decreasing |
| Epinephelus malabaricus | Malabar Grouper | Bony fishes | Marine | LC | Unchanged | Decreasing |
| Epinephelus marginatus | Dusky Grouper | Bony fishes | Marine | VU | Unchanged | Decreasing |
| Epinephelus tukula | Potato Grouper | Bony fishes | Marine | LC | Unchanged | Unknown |
| Erignathus barbatus | Bearded Seal | Marine Mammals | Marine, Terrestrial | LC | Unchanged | Unknown |
| Eschrichtius robustus | Gray Whale | Marine Mammals | Marine | LC | Improved | Stable |
| Esox lucius | Northern Pike | Bony fishes | Freshwater | LC | Unchanged | Stable |
| Esox masquinongy | Muskellunge | Bony fishes | Freshwater | LC | Unchanged | Stable |
| Eubalaena australis | Southern Right Whale | Marine Mammals | Marine | LC | Improved | Unknown |
| Eubalaena glacialis | North Atlantic Right Whale | Marine Mammals | Marine | EN | Unchanged | Decreasing |
| Eubalaena japonica | North Pacific Right Whale | Marine Mammals | Marine | EN | Unchanged | Unknown |
| Eumetopias jubatus | Steller Sea Lion | Marine Mammals | Marine, Terrestrial | NT | Improved | Increasing |
| Falco rusticolus | Gyrfalcon | Birds | Marine, Terrestrial | LC | Unchanged | Stable |
| Feresa attenuata | Pygmy Killer whale | Marine Mammals | Marine | LC | Unchanged | Unknown |
| Gadus morhua | Atlantic Cod | Bony fishes | Marine | VU | Unchanged | Unknown |
| Galeocerdo cuvier | Tiger Shark | Sharks and rays | Marine | NT | Unchanged | Decreasing |
| Gavialis gangeticus | Gharial | Reptiles | Freshwater, Terrestrial | CR | Declined | Increasing |
| Geranoaetus melanoleucus | Black-chested Buzzard-eagle | Birds | Terrestrial | LC | Unchanged | Stable |
| Grampus griseus | Risso's Dolphin | Marine Mammals | Marine | LC | Unchanged | Unknown |
| Gymnogyps californianus | California Condor | Birds | Terrestrial | CR | Unchanged | Increasing |
| Gymnosarda unicolor | Dogtooth Tuna | Bony fishes | Marine | LC | Unchanged | Unknown |
| Gymnura altavela | Spiny Butterfly Ray | Sharks and rays | Marine | VU | Unchanged | Decreasing |
| Gypaetus barbatus | Bearded Vulture | Birds | Terrestrial | NT | Unchanged | Decreasing |
| Gypohierax angolensis | Palm-nut Vulture | Birds | Freshwater, Terrestrial | LC | Unchanged | Stable |
| Gyps africanus | White-backed Vulture | Birds | Freshwater, Terrestrial | CR | Declined | Decreasing |
| Gyps bengalensis | White-rumped Vulture | Birds | Terrestrial | CR | Unchanged | Decreasing |
| Gyps coprotheres | Cape Vulture | Birds | Terrestrial | EN | Declined | Decreasing |
| Gyps fulvus | Griffon Vulture | Birds | Terrestrial | LC | Unchanged | Increasing |
| Gyps himalayensis | Himalayan Griffon | Birds | Terrestrial | NT | Unchanged | Stable |
| Gyps indicus | Indian Vulture | Birds | Terrestrial | CR | Unchanged | Decreasing |
| Gyps rueppelli | Rüppell's Vulture | Birds | Terrestrial | CR | Declined | Decreasing |
| Gyps tenuirostris | Slender-billed Vulture | Birds | Terrestrial | CR | Unchanged | Decreasing |
| Haliaeetus albicilla | White-tailed Sea-eagle | Birds | Marine, Freshwater, Terrestrial | LC | Improved | Increasing |
| Haliaeetus leucocephalus | Bald Eagle | Birds | Marine, Freshwater, Terrestrial | LC | Unchanged | Increasing |
| Haliaeetus leucogaster | White-bellied Sea-eagle | Birds | Marine, Freshwater, Terrestrial | LC | Unchanged | Decreasing |
| Haliaeetus leucoryphus | Pallas's Fish-eagle | Birds | Freshwater, Terrestrial | EN | Unchanged | Decreasing |
| Haliaeetus pelagicus | Steller's Sea-eagle | Birds | Marine, Freshwater, Terrestrial | VU | Unchanged | Decreasing |
| Haliaeetus sanfordi | Sanford's Sea-eagle | Birds | Marine, Freshwater, Terrestrial | VU | Unchanged | Decreasing |
| Haliaeetus vocifer | African Fish-eagle | Birds | Marine, Freshwater, Terrestrial | LC | Unchanged | Stable |
| Haliaeetus vociferoides | Madagascar Fish-eagle | Birds | Marine, Freshwater, Terrestrial | CR | Unchanged | Decreasing |
| Halichoerus grypus | Grey Seal | Marine Mammals | Marine, Terrestrial | LC | Unchanged | Increasing |
| Hamirostra melanosternon | Black-breasted Buzzard | Birds | Terrestrial | LC | Unchanged | Decreasing |
| Harpia harpyja | Harpy Eagle | Birds | Terrestrial | NT | Unchanged | Decreasing |
| Harpyopsis novaeguineae | Papuan Eagle | Birds | Terrestrial | VU | Unchanged | Decreasing |
| Hemipristis elongata | Fossil Shark | Sharks and rays | Marine | VU | Unchanged | Decreasing |
| Hexanchus griseus | Bluntnose Sixgill Shark | Sharks and rays | Marine | NT | Unchanged | Unknown |
| Hippoglossus hippoglossus | Atlantic Halibut | Bony fishes | Marine | EN | Unchanged | Unknown |
| Histriophoca fasciata | Ribbon Seal | Marine Mammals | Marine | LC | Unchanged | Unknown |
| Hucho hucho | Danube Salmon | Bony fishes | Freshwater | EN | Unchanged | Unknown |
| Hucho taimen | Siberian Taimen | Bony fishes | Freshwater | VU | Unchanged | Decreasing |
| Huso dauricus | Kaluga | Bony fishes | Marine, Freshwater | CR | Declined | Decreasing |
| Huso huso | Beluga | Bony fishes | Marine, Freshwater | CR | Declined | Decreasing |
| Hydrurga leptonyx | Leopard Seal | Marine Mammals | Marine, Terrestrial | LC | Unchanged | Unknown |
| Hyperoodon planifrons | Southern Bottlenose Whale | Marine Mammals | Marine | LC | Unchanged | Unknown |
| Hyporthodus ergastularius | Sevenbar Grouper | Bony fishes | Marine | NT | Declined | Decreasing |
| Hyporthodus mystacinus | Misty Grouper | Bony fishes | Marine | LC | Unchanged | Unknown |
| Ictalurus punctatus | Channel Catfish | Bony fishes | Freshwater | LC | Unchanged | Increasing |
| Ictinaetus malaiensis | Black Eagle | Birds | Terrestrial | LC | Unchanged | Decreasing |
| Istiophorus platypterus | Sailfish | Bony fishes | Marine | LC | Unchanged | Unknown |
| Isurus oxyrinchus | Shortfin Mako | Sharks and rays | Marine | EN | Unchanged | Decreasing |
| Isurus paucus | Longfin Mako | Sharks and rays | Marine | EN | Unchanged | Decreasing |
| Kajikia albida | White Marlin | Bony fishes | Marine | VU | Unchanged | Decreasing |
| Kajikia audax | Striped Marlin | Bony fishes | Marine | NT | Unchanged | Decreasing |
| Ketupa flavipes | Tawny Fish-owl | Birds | Freshwater, Terrestrial | LC | Unchanged | Decreasing |
| Ketupa ketupu | Buffy Fish-owl | Birds | Freshwater, Terrestrial | LC | Unchanged | Stable |
| Ketupa zeylonensis | Brown Fish-owl | Birds | Freshwater, Terrestrial | LC | Unchanged | Decreasing |
| Lagenodelphis hosei | Fraser's Dolphin | Marine Mammals | Marine | LC | Unchanged | Unknown |
| Lagenorhynchus acutus | Atlantic White-sided Dolphin | Marine Mammals | Marine | LC | Unchanged | Unknown |
| Lagenorhynchus albirostris | White-beaked Dolphin | Marine Mammals | Marine | LC | Unchanged | Unknown |
| Lagenorhynchus cruciger | Hourglass Dolphin | Marine Mammals | Marine | LC | Unchanged | Unknown |
| Lagenorhynchus obliquidens | Pacific White-sided Dolphin | Marine Mammals | Marine | LC | Unchanged | Unknown |
| Lamna ditropis | Salmon Shark | Sharks and rays | Marine | LC | Unchanged | Stable |
| Lamna nasus | Porbeagle | Sharks and rays | Marine | VU | Unchanged | Decreasing |
| Lampris guttatus | Opah | Bony fishes | Marine | LC | Unchanged | Unknown |
| Lates angustifrons | Tanganyika Lates | Bony fishes | Freshwater | EN | Unchanged | Decreasing |
| Lates niloticus | Nile Perch | Bony fishes | Freshwater | LC | Unchanged | Unknown |
| Latimeria chalumnae | Coelacanth | Bony fishes | Marine | CR | Unchanged | Unknown |
| Lepisosteus osseus | Longnose Gar | Bony fishes | Freshwater | LC | Unchanged | Stable |
| Leptonychotes weddellii | Weddell Seal | Marine Mammals | Marine, Terrestrial | LC | Unchanged | Unknown |
| Liasis olivaceus | Olive Python | Reptiles | Terrestrial | LC | Unchanged | Unknown |
| Lichia amia | Leerfish | Bony fishes | Marine | LC | Unchanged | Unknown |
| Lissodelphis borealis | Northern Right Whale Dolphin | Marine Mammals | Marine | LC | Unchanged | Unknown |
| Lobodon carcinophaga | Crabeater Seal | Marine Mammals | Marine, Terrestrial | LC | Unchanged | Unknown |
| Lophaetus occipitalis | Long-crested Eagle | Birds | Freshwater, Terrestrial | LC | Unchanged | Increasing |
| Lutjanus cyanopterus | Cubera Snapper | Bony fishes | Marine | VU | Unchanged | Decreasing |
| Lycalopex culpaeus | Culpeo | Terrestrial mammals | Terrestrial | LC | Unchanged | Stable |
| Lycaon pictus | African Wild Dog | Terrestrial mammals | Terrestrial | EN | Unchanged | Decreasing |
| Lynx lynx | Eurasian Lynx | Terrestrial mammals | Terrestrial | LC | Unchanged | Stable |
| Lynx pardinus | Iberian lynx | Terrestrial mammals | Terrestrial | EN | Improved | Increasing |
| Lynx rufus | Bobcat | Terrestrial mammals | Terrestrial | LC | Unchanged | Stable |
| Maccullochella peelii | Murray River Cod | Bony fishes | Freshwater | CR | Unchanged | Unknown |
| Makaira nigricans | Blue Marlin | Bony fishes | Marine | VU | Unchanged | Decreasing |
| Mecistops cataphractus | Slender-snouted Crocodile | Reptiles | Freshwater, Terrestrial | CR | Unchanged | Decreasing |
| Megalops atlanticus | Tarpon | Bony fishes | Marine | VU | Unchanged | Decreasing |
| Megaptera novaeangliae | Humpback Whale | Marine Mammals | Marine | LC | Improved | Increasing |
| Megatriorchis doriae | Doria's Goshawk | Birds | Terrestrial | NT | Unchanged | Decreasing |
| Melanosuchus niger | Black Caiman | Reptiles | Freshwater, Terrestrial | NT | Improved | Unknown |
| Milvus milvus | Red Kite | Birds | Terrestrial | NT | Declined | Decreasing |
| Mirounga angustirostris | Northern Elephant Seal | Marine Mammals | Marine, Terrestrial | LC | Unchanged | Increasing |
| Mirounga leonina | Southern Elephant Seal | Marine Mammals | Marine, Terrestrial | LC | Unchanged | Stable |
| Mitsukurina owstoni | Goblin Shark | Sharks and rays | Marine | LC | Unchanged | Unknown |
| Monachus monachus | Mediterranean Monk Seal | Marine Mammals | Marine, Terrestrial | EN | Unchanged | Increasing |
| Monodon monoceros | Narwhal | Marine Mammals | Marine | LC | Unchanged | Unknown |
| Morelia amethistina | Amethystine Python | Reptiles | Terrestrial | LC | Unchanged | Stable |
| Morelia bredli | Centralian Carpet Python | Reptiles | Terrestrial | LC | Unchanged | Stable |
| Morelia spilota | Western Australian Carpet Python | Reptiles | Terrestrial | LC | Unchanged | Decreasing |
| Morone saxatilis | Striped Bass | Bony fishes | Marine, Freshwater | LC | Unchanged | Unknown |
| Morphnarchus princeps | Barred Hawk | Birds | Terrestrial | LC | Unchanged | Decreasing |
| Morphnus guianensis | Crested Eagle | Birds | Terrestrial | NT | Unchanged | Decreasing |
| Mycteroperca bonaci | Black Grouper | Bony fishes | Marine | NT | Unchanged | Decreasing |
| Mycteroperca jordani | Gulf Grouper | Bony fishes | Marine | EN | Unchanged | Decreasing |
| Nebrius ferrugineus | Tawny Nurse Shark | Sharks and rays | Marine | VU | Unchanged | Decreasing |
| Necrosyrtes monachus | Hooded Vulture | Birds | Freshwater, Terrestrial | CR | Declined | Decreasing |
| Negaprion acutidens | Sharptooth Lemon Shark | Sharks and rays | Marine | VU | Unchanged | Decreasing |
| Negaprion brevirostris | Lemon Shark | Sharks and rays | Marine, Freshwater | NT | Unchanged | Unknown |
| Neofelis nebulosa | Clouded Leopard | Terrestrial mammals | Terrestrial | VU | Unchanged | Decreasing |
| Neomonachus schauinslandi | Hawaiian Monk Seal | Marine Mammals | Marine, Terrestrial | EN | Unchanged | Decreasing |
| Neophoca cinerea | Australian Sea Lion | Marine Mammals | Marine, Terrestrial | EN | Declined | Decreasing |
| Neophron percnopterus | Egyptian Vulture | Birds | Freshwater, Terrestrial | EN | Declined | Decreasing |
| Nesasio solomonensis | Fearful Owl | Birds | Terrestrial | VU | Unchanged | Decreasing |
| Ninox strenua | Powerful Owl | Birds | Freshwater, Terrestrial | LC | Unchanged | Stable |
| Nisaetus cirrhatus | Changeable Hawk-eagle | Birds | Terrestrial | LC | Unchanged | Decreasing |
| Nisaetus floris | Flores Hawk-eagle | Birds | Terrestrial | CR | Unchanged | Decreasing |
| Nisaetus nipalensis | Mountain Hawk-eagle | Birds | Terrestrial | LC | Unchanged | Decreasing |
| Nisaetus philippensis | North Philippine Hawk-eagle | Birds | Terrestrial | EN | Unchanged | Decreasing |
| Odobenus rosmarus | Walrus | Marine Mammals | Marine, Terrestrial | VU | Unchanged | Unknown |
| Odontaspis ferox | Smalltooth Sand Tiger | Sharks and rays | Marine | VU | Unchanged | Decreasing |
| Ommatophoca rossii | Ross Seal | Marine Mammals | Marine, Terrestrial | LC | Unchanged | Unknown |
| Ophichthus ophis | Spotted Snake Eel | Bony fishes | Marine | LC | Unchanged | Unknown |
| Ophisurus serpens | Serpent Eel | Bony fishes | Marine | LC | Unchanged | Unknown |
| Orcaella brevirostris | Irrawaddy Dolphin | Marine Mammals | Marine, Freshwater | EN | Unchanged | Decreasing |
| Orectolobus maculatus | Spotted Wobbegong | Sharks and rays | Marine | LC | Unchanged | Unknown |
| Osteolaemus tetraspis | African Dwarf Crocodile | Reptiles | Freshwater, Terrestrial | VU | Unchanged | Unknown |
| Otaria byronia | South American Sea Lion | Marine Mammals | Marine, Terrestrial | LC | Unchanged | Stable |
| Pagophilus groenlandicus | Harp Seal | Marine Mammals | Marine | LC | Unchanged | Increasing |
| Pandion haliaetus | Osprey | Birds | Marine, Freshwater, Terrestrial | LC | Unchanged | Increasing |
| Panthera leo | Lion | Terrestrial mammals | Terrestrial | VU | Unchanged | Decreasing |
| Panthera onca | Jaguar | Terrestrial mammals | Terrestrial | NT | Unchanged | Decreasing |
| Panthera pardus | Leopard | Terrestrial mammals | Terrestrial | VU | Declined | Decreasing |
| Panthera tigris | Tiger | Terrestrial mammals | Terrestrial | EN | Unchanged | Decreasing |
| Panthera uncia | Snow Leopard | Terrestrial mammals | Terrestrial | VU | Unchanged | Decreasing |
| Parahyaena brunnea | Brown Hyaena | Terrestrial mammals | Terrestrial | NT | Unchanged | Stable |
| Pardofelis marmorata | Marbled Cat | Terrestrial mammals | Terrestrial | NT | Unchanged | Decreasing |
| Peponocephala electra | Melon-headed Whale | Marine Mammals | Marine | LC | Unchanged | Unknown |
| Petrus rupestris | Red Steenbras | Bony fishes | Marine | EN | Unchanged | Decreasing |
| Phalcoboenus australis | Striated Caracara | Birds | Marine, Terrestrial | NT | Unchanged | Stable |
| Phoca largha | Spotted Seal | Marine Mammals | Marine, Terrestrial | LC | Unchanged | Unknown |
| Phoca vitulina | Harbor Seal | Marine Mammals | Marine, Freshwater, Terrestrial | LC | Unchanged | Unknown |
| Phocarctos hookeri | New Zealand Sea Lion | Marine Mammals | Marine, Terrestrial | EN | Declined | Decreasing |
| Phocoena phocoena | Harbour Porpoise | Marine Mammals | Marine | LC | Unchanged | Unknown |
| Phocoenoides dalli | Dall's Porpoise | Marine Mammals | Marine | LC | Unchanged | Unknown |
| Physeter macrocephalus | Sperm Whale | Marine Mammals | Marine | VU | Unchanged | Unknown |
| Pithecophaga jefferyi | Philippine Eagle | Birds | Terrestrial | CR | Unchanged | Decreasing |
| Plesiobatis daviesi | Giant Stingaree | Sharks and rays | Marine | LC | Unchanged | Unknown |
| Polemaetus bellicosus | Martial Eagle | Birds | Freshwater, Terrestrial | EN | Declined | Decreasing |
| Polydactylus quadrifilis | Giant African threadfin | Bony fishes | Marine | LC | Unchanged | Stable |
| Prionace glauca | Blue Shark | Sharks and rays | Marine | NT | Unchanged | Unknown |
| Pristis pectinata | Smalltooth Sawfish | Sharks and rays | Marine | CR | Unchanged | Decreasing |
| Psephurus gladius | Chinese Paddlefish | Bony fishes | Marine, Freshwater | CR | Unchanged | Unknown |
| Pseudorca crassidens | False Kille Whale | Marine Mammals | Marine | NT | Unchanged | Unknown |
| Pseudotriakis microdon | False Catshark | Sharks and rays | Marine | LC | Unchanged | Unknown |
| Pteronura brasiliensis | Giant Otter | Terrestrial mammals | Freshwater, Terrestrial | EN | Unchanged | Decreasing |
| Ptyas carinata | Keeled Rat Snake | Reptiles | Terrestrial | LC | Unchanged | Decreasing |
| Puma concolor | Puma | Terrestrial mammals | Terrestrial | LC | Unchanged | Decreasing |
| Pusa caspica | Caspian Seal | Terrestrial mammals | Freshwater, Terrestrial | EN | Unchanged | Unknown |
| Pusa hispida | Ringed Seal | Marine Mammals | Marine, Freshwater, Terrestrial | LC | Unchanged | Unknown |
| Pusa sibirica | Baikal Seal | Terrestrial mammals | Freshwater, Terrestrial | LC | Unchanged | Stable |
| Python bivittatus | Burmese Python | Reptiles | Terrestrial | VU | Unchanged | Decreasing |
| Python reticulatus | Reticulated Python | Reptiles | Terrestrial | LC | Unchanged | Unknown |
| Regalecus russelii | Oarfish | Bony fishes | Marine | LC | Unchanged | Unknown |
| Rhynchobatus luebberti | African Wedgefish | Sharks and rays | Marine | EN | Unchanged | Decreasing |
| Rostroraja alba | White Skate | Sharks and rays | Marine | EN | Unchanged | Decreasing |
| Ruvettus pretiosus | Oilfish | Bony fishes | Marine | LC | Unchanged | Stable |
| Sagittarius serpentarius | Secretarybird | Birds | Terrestrial | VU | Unchanged | Decreasing |
| Salmo marmoratus | NA | Bony fishes | Freshwater | LC | Unchanged | Decreasing |
| Sander lucioperca | Pike-perch | Bony fishes | Freshwater | LC | Unchanged | Unknown |
| Sarcogyps calvus | Red-headed Vulture | Birds | Terrestrial | CR | Declined | Decreasing |
| Sarcoramphus papa | King Vulture | Birds | Terrestrial | LC | Unchanged | Decreasing |
| Sciades parkeri | Gillbacker Sea Catfish | Bony fishes | Marine, Freshwater | VU | Unchanged | Decreasing |
| Scomberomorus commerson | Narrow-barred Spanish Mackerel | Bony fishes | Marine | NT | Unchanged | Decreasing |
| Scotopelia peli | Pel's Fishing-owl | Birds | Marine, Freshwater, Terrestrial | LC | Unchanged | Decreasing |
| Seriola dumerili | Greater Amberjack | Bony fishes | Marine | LC | Unchanged | Unknown |
| Seriola hippos | Samson Fish | Bony fishes | Marine | LC | Unchanged | Unknown |
| Seriola lalandi | Yellowtail Amberjack | Bony fishes | Marine | LC | Unchanged | Stable |
| Seriola rivoliana | Longfin Yellowtail | Bony fishes | Marine | LC | Unchanged | Stable |
| Silurus asotus | Amur catfish | Bony fishes | Freshwater | LC | Unchanged | Unknown |
| Silurus glanis | Wels Catfish | Bony fishes | Freshwater | LC | Unchanged | Unknown |
| Somniosus microcephalus | Greenland Shark | Sharks and rays | Marine | NT | Unchanged | Unknown |
| Sousa chinensis | Indo-Pacific Humpbacked Dolphin | Marine Mammals | Marine, Freshwater | VU | Unchanged | Decreasing |
| Sousa teuszii | Atlantic Humpbacked Dolphin | Marine Mammals | Marine, Freshwater | CR | Unchanged | Decreasing |
| Sphyraena afra | Guinean Barracuda | Bony fishes | Marine | LC | Unchanged | Unknown |
| Sphyraena barracuda | Great Barracuda | Bony fishes | Marine | LC | Unchanged | Unknown |
| Sphyrna lewini | Scalloped Hammerhead | Sharks and rays | Marine | CR | Unchanged | Unknown |
| Sphyrna mokarran | Great Hammerhead | Sharks and rays | Marine | CR | Unchanged | Decreasing |
| Sphyrna zygaena | Smooth Hammerhead | Sharks and rays | Marine | VU | Unchanged | Decreasing |
| Spizaetus ornatus | Ornate Hawk-eagle | Birds | Terrestrial | NT | Declined | Decreasing |
| Spizaetus tyrannus | Black Hawk-eagle | Birds | Freshwater, Terrestrial | LC | Unchanged | Decreasing |
| Squatina squatina | Angelshark | Sharks and rays | Marine | CR | Unchanged | Decreasing |
| Stenella attenuata | Pantropical Spotted Dolphin | Marine Mammals | Marine | LC | Unchanged | Unknown |
| Stenella coeruleoalba | Striped Dolphin | Marine Mammals | Marine | LC | Unchanged | Unknown |
| Steno bredanensis | Rough-toothed Dolphin | Marine Mammals | Marine | LC | Unchanged | Unknown |
| Stephanoaetus coronatus | Crowned Eagle | Birds | Freshwater, Terrestrial | NT | Unchanged | Decreasing |
| Stereolepis gigas | Giant Sea Bass | Bony fishes | Marine | CR | Unchanged | Unknown |
| Strix leptogrammica | Brown Wood-owl | Birds | Terrestrial | LC | Unchanged | Decreasing |
| Strix nebulosa | Great Grey Owl | Birds | Freshwater, Terrestrial | LC | Unchanged | Increasing |
| Strix seloputo | Spotted Wood-owl | Birds | Terrestrial | LC | Unchanged | Stable |
| Taeniurops meyeni | Blotched Fantail Ray | Sharks and rays | Marine | VU | Unchanged | Decreasing |
| Terathopius ecaudatus | Bateleur | Birds | Terrestrial | NT | Unchanged | Decreasing |
| Tetrapturus belone | Mediterranean Shortbill Spearfish | Bony fishes | Marine | LC | Unchanged | Stable |
| Tetrapturus pfluegeri | Longbill Spearfish | Bony fishes | Marine | LC | Unchanged | Stable |
| Thunnus alalunga | Albacore Tuna | Bony fishes | Marine | NT | Unchanged | Decreasing |
| Thunnus albacares | Yellowfin Tuna | Bony fishes | Marine | NT | Declined | Decreasing |
| Thunnus obesus | Bigeye Tuna | Bony fishes | Marine | VU | Unchanged | Decreasing |
| Thunnus orientalis | Pacific Bluefin Tuna | Bony fishes | Marine | VU | Unchanged | Decreasing |
| Thunnus thynnus | Atlantic Bluefin Tuna | Bony fishes | Marine | EN | Unchanged | Decreasing |
| Tomistoma schlegelii | False Gharial | Reptiles | Freshwater, Terrestrial | VU | Unchanged | Decreasing |
| Torgos tracheliotos | Lappet-faced Vulture | Birds | Terrestrial | EN | Declined | Decreasing |
| Totoaba macdonaldi | Totoaba | Bony fishes | Marine, Freshwater | CR | Unchanged | Decreasing |
| Trachipterus trachypterus | Mediterranean Dealfish | Bony fishes | Marine | LC | Unchanged | Unknown |
| Trigonoceps occipitalis | White-headed Vulture | Birds | Terrestrial | CR | Declined | Decreasing |
| Tursiops truncatus | Common Bottlenose Dolphin | Marine Mammals | Marine | LC | Unchanged | Unknown |
| Varanus exanthematicus | Savannah Monitor | Reptiles | Terrestrial | LC | Unchanged | Unknown |
| Varanus komodoensis | Komodo Dragon | Reptiles | Terrestrial | VU | Unchanged | Unknown |
| Varanus salvadorii | Crocodile Monitor | Reptiles | Terrestrial | LC | Unchanged | Unknown |
| Vultur gryphus | Andean Condor | Birds | Terrestrial | NT | Unchanged | Decreasing |
| Xiphias gladius | Swordfish | Bony fishes | Marine | LC | Unchanged | Decreasing |
| Zalophus californianus | Californian Sea Lion | Marine Mammals | Marine, Terrestrial | LC | Unchanged | Increasing |
| Zalophus wollebaeki | Galápagos Sea Lion | Marine Mammals | Marine, Terrestrial | EN | Declined | Decreasing |
| Ziphius cavirostris | Cuvier's Beaked Whale | Marine Mammals | Marine | LC | Unchanged | Unknown |
